# The Whisper of the Follicle: A Systematic Review of Micro Ribonucleic Acids as Predictors of Oocyte Quality and In Vitro Fertilization Outcomes

**DOI:** 10.3390/cells14110787

**Published:** 2025-05-27

**Authors:** Charalampos Voros, Antonia Varthaliti, Diamantis Athanasiou, Despoina Mavrogianni, Anthi-Maria Papahliou, Kyriakos Bananis, Aristotelis-Marios Koulakmanidis, Antonia Athanasiou, Aikaterini Athanasiou, Constantinos G. Zografos, Athanasios Gkirgkinoudis, Maria Anastasia Daskalaki, Dimitris Mazis Kourakos, Dimitrios Vaitsis, Ioannis Papapanagiotou, Marianna Theodora, Panagiotis Antsaklis, Dimitrios Loutradis, Georgios Daskalakis

**Affiliations:** 1 1st Department of Obstetrics and Gynecology, ‘Alexandra’ General Hospital, National and Kapodistrian University of Athens, 80 Vasilissis Sofias Avenue, 11528 Athens, Greece; antonia.varthaliti@hotmail.com (A.V.); depy.mavrogianni@yahoo.com (D.M.); anthipapahliou@gmail.com (A.-M.P.); aristoteliskoulak@gmail.com (A.-M.K.); tgkirgki@gmail.com (A.G.); md181341@students.euc.ac.cy (M.A.D.); gpapamd@hotmail.com (I.P.); martheodr@gmail.com (M.T.); panosant@gmail.com (P.A.); gdaskalakis@yahoo.com (G.D.); 2 King’s College Hospitals NHS Foundation Trust, London SE5 9RS, UK; diamathan16@gmail.com (D.A.); antoathan16@gmail.com (A.A.); aikaterathan16@gmail.com (A.A.); 3 IVF Athens Reproduction Center V. Athanasiou, 15123 Maroussi, Greece; kyriakos.bananis@nhs.net; 4 2nd Surgical Department, General Hospital of Athens “LAIKO”, 11527 Athens, Greece; koszogra92@hotmail.com; 5 Rea Maternity Hospital S.A., Avenue Siggrou 383 & Pentelis 17, P. Faliro, 17564 Athens, Greece; mazisdimitris@gmail.com; 6 Athens Medical School, National and Kapodistrian University of Athens, 15772 Athens, Greece; vaitsisdim@gmail.com (D.V.); loutradi@otenet.gr (D.L.); 7 Assisted Reproduction Unit, Fertility Institute, Paster 15, 11528 Athens, Greece

**Keywords:** microRNAs, follicular fluid, PCOS, in vitro fertilization, biomarkers, oocyte quality, embryonic development, granulosa cells, reproductive outcomes

## Abstract

**Background:** MicroRNAs (miRNAs) in follicular fluid (FF) are being recognized as important regulators of ovarian function and biomarkers of reproductive success. This systematic analysis investigates FF-derived miRNAs and their relationship to polycystic ovarian syndrome (PCOS) and in vitro fertilization (IVF) outcomes. **Methods:** Following PRISMA recommendations, 21 original papers were included that looked at miRNA expression in FF or granulosa cells from women undergoing IVF, with or without PCOS. The study design, miRNA profiling methodologies, IVF protocols, and clinical results were gathered and analyzed. **Results:** Across the investigations, 15 miRNAs were regularly implicated, including miR-132, miR-320, miR-222, miR-224, miR-146a, and miR-93. Downregulation of miR-132 and miR-320 was consistently detected in PCOS and associated with decreased steroidogenesis. Elevated miR-222 and miR-146a were linked to insulin resistance and follicular inflammation. In IVF, miR-202-5p and miR-224 were elevated in high-quality embryos and successful cycles, indicating that they have roles in granulosa cell proliferation and estrogen synthesis. MiRNA dysregulation was linked to critical pathways, such as PI3K/AKT, NF-κB, TGF-β, and WNT. **Conclusions:** Specific FF miRNAs are consistently linked to PCOS pathogenesis and IVF effectiveness. Their use into noninvasive biomarker panels could improve embryonic selection and personalized reproductive care.

## 1. Introduction

Polycystic ovarian syndrome (PCOS) is a multifactorial endocrine condition that is one of the most common causes of female infertility globally. It affects almost one in every ten women of reproductive age and is associated with a variety of reproductive and metabolic problems [1].

These include persistent anovulation, hyperandrogenism, insulin resistance, and polycystic ovarian morphology. A key aspect of PCOS-related infertility is the disturbance of folliculogenesis, which is the process by which ovarian follicles mature and finally release a viable egg. This process is closely controlled at the molecular level by a complex network of hormonal signals, intercellular communication, and gene expression regulation, as well as epigenetic mechanisms [2].

MicroRNAs (miRNAs) have emerged as important post-transcriptional modulators of gene expression, with far-reaching ramifications in reproductive biology. MiRNAs are short (~22 nucleotides) non-coding RNAs that regulate gene expression by binding to complementary sequences inside the 3′ untranslated regions (3′UTRs) of target mRNAs [3]. MiRNAs bind and cause either translational repression or mRNA destruction, depending on the degree of sequence complementarity and the biological environment [4]. Each miRNA can regulate numerous target genes, and individual mRNAs can be co-regulated by multiple miRNAs, resulting in complex regulatory networks that keep cells stable [5].

MiRNAs are highly expressed in granulosa cells, theca cells, and oocytes in the ovary, and their levels change dynamically during follicular development [6]. Their expressions vary in response to hormonal stimuli, like follicle-stimulating hormone (FSH) and luteinizing hormone (LH), which regulate their synthesis through DROSHA- and DICER-dependent pathways [7]. Functionally, ovarian miRNAs regulate a variety of activities required for effective reproduction, including granulosa cell proliferation and differentiation, steroid hormone biosynthesis, cumulus expansion, luteinization, and apoptosis resistance [8].

These effects are primarily mediated by interactions with numerous highly conserved intracellular signaling pathways, for example:

- MiRNAs, such as miR-93 and miR-21, affect the PI3K/AKT pathway, which is required for follicular activation and survival and regulates components such as PTEN and FOXO1. Activation of this mechanism promotes cell survival while inhibiting granulosa cell death [9];
- The WNT/β-catenin-signaling cascade regulates follicle maturation, cumulus expansion, and ovulation. MiRNAs, such as miR-766-3p and let-7 family members, have been linked to influencing this pathway by targeting WNT ligands and downstream transcription factors [10];
- The TGF-β/Smad pathway regulates granulosa cell proliferation and extracellular matrix remodeling. MiRNAs, such as miR-224, influence this pathway by targeting SMAD4 and regulating aromatase (CYP19A1) expression, which influences estradiol synthesis [11];
- The MAPK/ERK pathway is important in LH-induced ovulatory signaling. Several miRNAs, including miR-132, have been found to impact ERK activation and downstream transcriptional responses required for luteinization [12];
- In PCOS, the NF-κB pathway, which regulates inflammation, is overactivated, leading to a proinflammatory follicular milieu. MiR-146a inhibits this pathway by targeting adaptor proteins, such TRAF6 and IRAK1, which affect cytokine release and granulosa cell viability [13].

MiRNAs act as molecular integrators of endocrine signals and intracellular signaling, regulating ovarian cell behavior and fate. Their dysregulation, as seen in pathological diseases, such as PCOS, can upset this delicate balance, resulting in abnormal folliculogenesis, faulty steroidogenesis, impaired oocyte maturation, and reduced fertility [14].

Follicular fluid (FF) is the biological medium that surrounds the oocyte within the antral follicle and plays important roles in oocyte growth and maturation [15]. It is a complex and dynamic milieu composed of plasma transudate and secretions from surrounding follicular cells, particularly granulosa and theca cells [16]. FF contains steroidal hormones (e.g., estradiol, progesterone, and testosterone), gonadotropins (e.g., follicle-stimulating hormone (FSH) and luteinizing hormone (LH)), cytokines (e.g., IL-6 and TNF-α), metabolites, proteins, and non-coding RNAs (miRNAs) [17]. The molecular composition of FF offers a real-time biochemical snapshot of the follicle’s health, functioning, and developmental potential.

Among its various contents, miRNAs have received substantial attention as essential regulators in the FF microenvironment. These tiny, non-coding RNAs are released into FF either freely or encased in extracellular vesicles (EVs), like exosomes and microvesicles [18]. Exosomes are endosome-derived nanovesicles (30–150 nm) that facilitate intercellular communication by transferring molecular cargo, such as miRNAs, messenger RNAs (mRNAs), and proteins, from donor to recipient cells. Exosomes released by granulosa cells, cumulus cells, and even the oocyte allow for bidirectional communication, which is required for synchronized follicular development [19].

Encapsulating miRNAs within EVs not only keeps them stable in the extracellular environment by protecting them from ribonuclease degradation but also allows for targeted distribution to specific recipient cells via receptor-mediated uptake or membrane fusion [20]. This mechanism allows miRNAs to play both autocrine and paracrine roles, regulating granulosa cell proliferation, oocyte cytoplasmic and nuclear maturations, cumulus expansion, and even early embryonic development [21].

Several miRNAs identified in FF have been associated with important features of oocyte quality and IVF results. For example, miR-202-5p regulates luteinizing hormone/choriogonadotropin receptor (LHCGR) expression and has been linked to increased fertilization rates. MiR-224 enhances estradiol production by targeting Smad4 and increasing aromatase (CYP19A1) activity [22]. High levels of proinflammatory miRNAs, such as miR-155 and miR-146a, have been associated with oxidative stress and poor oocyte competence, especially in women with polycystic ovarian syndrome (PCOS) [23]. Importantly, the miRNA content of FF not only reflects local follicular health but also may influence future reproductive outcomes. It has been postulated that altered FF miRNA profiles lead to abnormal oocyte–granulosa cell communication, insufficient luteinization, and poor follicular remodeling [24]. These alterations are especially important in pathological disorders, like PCOS, which frequently exhibit follicular stoppage, hyperandrogenism, and chronic low-grade inflammation [25]. In such cases, the dysregulated production and absorption of EV-encapsulated miRNAs may worsen the intrafollicular imbalance and reduce reproductive capacity even further.

Despite the rising body of evidence relating FF miRNAs to ovarian physiology and reproductive success, no previous systematic review has thoroughly analyzed the molecular and clinical importance of these short RNAs in both PCOS and IVF [26]. The existing literature is fragmented, with individual research reporting miRNA profiles, target genes, and signaling pathways but no comprehensive framework linking these findings to clinical outcomes and diagnostic relevance [27]. This systematic review seeks to close that gap by critically reviewing and combining current information from original research that measured miRNA expression in FF or granulosa cells in women undergoing IVF, with or without PCOS. We identify consistently dysregulated miRNAs; investigate their validated gene targets and associated signaling pathways, such as PI3K/AKT, TGF-β, NF-κB, and WNT; and assess their link with critical reproductive characteristics, including oocyte quality, embryonic development, and clinical pregnancy.

MiRNAs produced from follicular fluid offer a more accurate and temporally pertinent perspective of the ovarian environment compared to those of systemic fluids, such as serum or plasma. An improved biospecimen for assessing oocyte competence and predicting IVF outcomes, follicular fluid captures local paracrine and autocrine interactions among granulosa cells, cumulus cells, and the oocyte. Although more accessible, circulating miRNAs may indicate systemic impacts and lack the specificity of intrafollicular signaling patterns.

The question of whether miRNA dysregulation is a primary contributor to the pathophysiology of PCOS or a resultant impact of its metabolic and endocrine problems remains a key unresolved research issue. Although cross-sectional studies indicate altered miRNA expression in PCOS, longitudinal and functional studies are necessary to elucidate causality and determine whether specific miRNAs actively contribute to follicular arrest, hyperandrogenism, and chronic inflammation or if they are merely a consequence of these dysregulated processes.

This systematic analysis provides a novel and comprehensive synthesis of contemporary discoveries regarding miRNAs specifically sourced from the follicular compartment, including follicular fluid, granulosa cells, and extracellular vesicles, within the contexts of PCOS and IVF. In contrast to previous studies that prioritize circulating biomarkers or fail to distinguish between sample sources, our research concentrates on the local ovarian environment, which directly influences oocyte maturation and embryonic development. Furthermore, we propose a novel classification of miRNAs according to their clinical significance (e.g., embryonic quality, implantation, and pregnancy), mechanistic targets (e.g., IGF1R, TRAF6, and CYP19A1), and regulatory pathways (e.g., PI3K/AKT, TGF-β/Smad, and NF-κB). This PRISMA-compliant synthesis identifies information deficiencies, highlights frequently reported miRNAs, and offers translational guidance for the advancement of biomarkers in ART.

## 2. Material and Methods

### 2.1. Protocol and Registration

This systematic review was designed and carried out in accordance with the Preferred Reporting Items for Systematic Reviews and Meta-Analyses (PRISMA) 2020 standards, which include transparency, completeness, and reproducibility when reporting evidence syntheses. The PRISMA methodology was closely adhered to throughout the review process, including the development of the study question, literature search, eligibility screening, and data extraction and synthesis.

A standardized methodology was created in advance to reduce prejudice and promote methodological rigor. This protocol established the review objectives, eligibility criteria, information sources, search strategy, data extraction fields, and risk-of-bias assessment procedures. To provide transparency and enable the public tracking of the methodological integrity, the protocol was prospectively registered in the International Prospective Registry of Systematic Reviews (PROSPERO), with the registration number CRD420251001390.

The registration record is publicly available and contains complete information about the review scope, including the research question formulated using the Population, Exposure, Comparator, and Outcome (PECO) structure, as well as the intended data synthesis methodologies. Any changes from the registration protocol were documented and disclosed in the final paper to ensure that systematic reviews in biomedical research followed the best standards.

### 2.2. Eligibility Criteria

Studies were considered as eligible if they examined miRNA expression in the follicular milieu during ART. Eligible samples included FF, GCs, or EVs produced from FF obtained from women undergoing IVF and who were either diagnosed with PCOS or did not have any endocrine/metabolic disorders. This review focused solely on human trials in which participants of reproductive age underwent COS and oocyte extraction as a part of ART regimens.

Only original research articles published in peer-reviewed journals were considered. Acceptable study designs included observational studies (case control, cross-sectional, or prospective cohort), as well as experimental studies that used ex vivo or in vitro cultures of IVF-derived GCs. A crucial inclusion requirement was miRNA detection using validated molecular platforms, such as RT-qPCR, microarrays, TaqMan-based assays, and NGS. Studies that used bioinformatic predictions without experimental validation of miRNA expressions were eliminated.

To be eligible, studies had to report links between miRNA expression and clinically relevant reproductive outcomes, like oocyte maturation, embryonic morphology or grading, blastocyst development, implantation, biochemical or clinical pregnancy, and hormonal profiles (e.g., E2, P4, T, LH, FSH, and AMH). Studies that focused primarily on miRNAs in serum, plasma, or non-follicular compartments were omitted, as were studies that did not report on IVF-related outcomes.

Articles published in English were evaluated, assuming full-text access was accessible. Exclusion criteria included reviews, systematic reviews, meta-analyses, editorials, letters, conference papers, case reports, animal experiments, and studies with insufficient methodological detail or outcome data. Studies containing mixed samples (e.g., pooled FF and serum) were omitted unless the FF results were reported separately.

Each publication was evaluated based on its clinical relevance to IVF and/or PCOS, methodological clarity, miRNA detection methods, and association with reproductive or molecular results. Only studies with extractable data on differentially expressed miRNAs and their relationship to IVF parameters were chosen for the final synthesis.

### 2.3. Information Resources and Search Strategy

A thorough and highly sensitive search method was developed to gather all the relevant material on miRNA expression in FF, GCs, and EVs in relation to IVF and/or PCOS. Three important biomedical databases—PubMed, Scopus, and WoS—were searched. These platforms were chosen because they provide complementary indexing of clinical, molecular biology, and translational research, as well as the capacity to provide organized and reproducible search queries that use Boolean logic, field tags, and filters.

To achieve extensive coverage of terminological changes, the search technique used both free-text keywords and regulated vocabulary (for example, MeSH terms in PubMed). Keywords and combinations included miRNA and microRNA; FF, GCs, or EVs; IVF, ART, and COS; and PCOS. These terms were searched in the titles, abstracts, and keywords. Search queries were tailored to the database syntax—for example, field codes, like “tiab” and “mh”, were used in PubMed, while TITLE-ABS-KEY was utilized in Scopus. To increase sensitivity, truncation operators (such as “microRNA*”) and phrasal searching were used.

The date range was unconstrained, spanning all the publications until 31 March 2025. There were no language filters used during the initial search phase; however, only studies published in English were retained during the screening. The search was revised twice before the manuscript completion to include the most recent evidence.

All the records were exported to EndNote X9, where both automated and human deduplication were performed. The unique citations were then put into a shared screening platform and reviewed by two independent investigators. Additional relevant papers were discovered by manually searching the reference lists of the listed publications and tracking forward citations using Google Scholar and WoS.

To reduce reporting bias and increase coverage, alerts were set up for PubMed and Scopus to monitor any newly published studies during the review process. Although the gray literature was excluded (e.g., preprints, conference abstracts, or non-peer-reviewed reports), all the included studies were published in peer-reviewed journals that were indexed in at least one of the chosen databases. The complete search strategy, which included the number of hits per query and the filtering process, was documented and saved. The PRISMA 2020 flow diagram in the results section visualizes the entire selection method, from the initial retrieval to the final inclusion.

### 2.4. Study Selection and Screening

After finishing the database search and reference harvesting, all the collected citations were imported into Zotero, an open-source reference management program. Zotero’s duplicate detection tool was used to find and integrate redundant records from multiple databases. Manual verification was also performed to ensure that studies with minor metadata differences were not incorrectly excluded. Once deduplication was complete, the curated reference set was exported and screened according to a preset two-phase process.

In the first phase (title and abstract screenings), two reviewers (Author 1 and Author 2) independently evaluated each reference for relevance, using the preset eligibility criteria outlined in Section 2.2. Studies were excluded at this stage if they were (i) irrelevant to the review question (e.g., studies not involving miRNAs, IVF, or PCOS); (ii) conducted in animal models or cell lines not derived from human ovarian tissue; (iii) focused on male infertility; (iv) reviews, editorials, conference abstracts, or case reports; or (v) investigated miRNAs in biological fluids other than FF (e.g., serum, plasma, and urine).

In the second phase (full-text screening), the complete texts of all the possibly relevant publications were retrieved and thoroughly reviewed by the same two reviewers. Screening was conducted separately, using a piloted form based on the PECO framework. Articles were included only if they (a) clearly investigated miRNA expressions in FF, GCs, or EVs and (b) presented data linking miRNAs to PCOS-related characteristics or IVF outcomes. When full-text access was not immediately accessible, the authors were contacted. Where information about the sample type or methodology was lacking or ambiguous, the procedures and extra materials were thoroughly evaluated to determine eligibility.

Disagreements were recorded during both rounds and resolved through discussion between the two reviewers. If no agreement was obtained, a third independent reviewer (Author 3) assessed the article and made the final decision. Because of the nature of narrative syntheses, the reviewers were not blinded to the publishing source or authors throughout the process; nonetheless, all the screening was completed solely based on eligibility criteria to reduce selection bias. The reason for each study’s exclusion at the full-text stage was documented and cataloged in accordance with PRISMA guidelines. This material is available upon request and can be delivered as a separate file if necessary.

**Figure 1:**
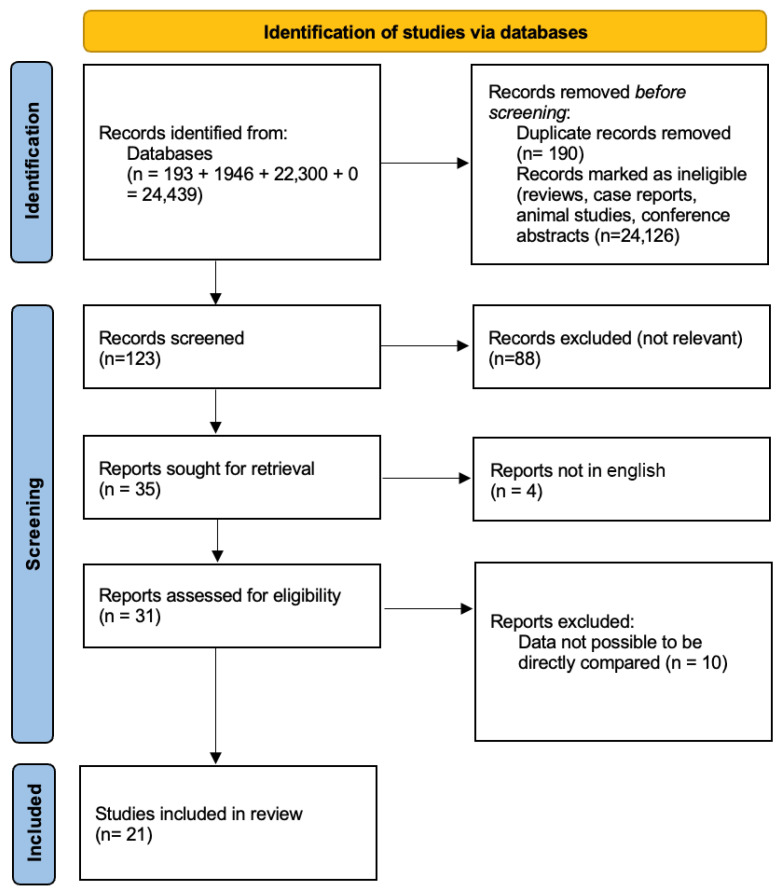
PRISMA 2020 flow diagram of the study selection process. This flow diagram depicts the step-by-step process of study identification, screening, eligibility assessment, and the final inclusion using the PRISMA 2020 technique. It provides a clear overview of the number of records found through database searches, duplicates deleted, records excluded at each stage, and the final studies reviewed (n = 21).

A total of 21 original studies passed the final inclusion criteria and were chosen for data extraction and analysis. These research studies come from various geographical locations, sample types (FF, GCs, and EVs), and miRNA-profiling platforms, but they all met the basic methodological standards for quality and relevance. The PRISMA 2020 flow diagram (Figure 1) graphically summarizes the study selection process by showing the number of records detected, screened, and excluded (with reasons) and included in the final analysis.

Table 1 contains the detailed eligibility structure that drove the study selection process for this systematic review. Criteria were developed using the PECO (population, exposure, comparator, and outcome) methodology to assure the inclusion of methodologically relevant and clinically useful studies. The target group was women of reproductive age, having IVF, either with a PCOS diagnosis or as normal ovulatory controls. Acceptable sample types included FF, GCs, and EVs produced from FF, allowing researchers to focus on the follicular milieu that is directly involved in oocyte maturation and competence.

To ensure analytical rigor, only original, peer-reviewed publications using validated miRNA detection technologies (e.g., RT-qPCR, microarrays, TaqMan assays, or NGS) were considered. The studies had to show a link between miRNA expression and IVF or PCOS outcomes, such as egg quality, embryonic grade, hormone levels, or pregnancy. Exclusion criteria were used to exclude reviews, animal studies, case reports, studies with inaccessible full texts, and research using non-follicular sample sources.

This organized strategy guaranteed that the final set of the included research was homogeneous in biological breadth while also diverse in methodology and outcome reporting, allowing for a meaningful synthesis. The criteria also enabled us to concentrate on the translational significance of miRNA biomarkers in human IVF and PCOS settings.

### 2.5. Data Extraction

Two reviewers (Author 1 and Author 2) extracted data independently, using a standardized and piloted extraction form designed expressly for this review. The form was created to collect crucial methodological, clinical, and molecular factors relevant to the review’s aims. Discrepancies in extracted data were handled through consensus or by a third reviewer (Author 3). To verify correctness, an impartial reviewer who was not involved in the screening phase evaluated a random subset of 20% of the extracted data.

The following information was systematically extracted from each included study: the first author, year of publication, country of origin, study design, total sample size, sample type (FF, GCs, or EVs), IVF protocol (e.g., agonist/antagonist COS regimen), PCOS diagnostic criteria (if applicable), miRNA detection method (e.g., RT-qPCR and NGS), and whether the study assessed targeted or global miRNA expression.

In addition, detailed information was collected on the differentially expressed miRNAs and their direction of change (up- or downregulation), verified or anticipated molecular targets (e.g., IGF1R, CYP19A1, and ESR1), and associated signaling pathways (e.g., PI3K/AKT, NF-κB, WNT, and TGF-β). When reported, correlations between miRNA expression and clinical outcomes, such as the oocyte maturity, embryonic quality, blastulation rate, implantation, and clinical pregnancy, were also identified. The study’s ability to offer quantifiable data (e.g., the fold change, *p*-value, log2FC, and AUC) for each reported miRNA was especially important. If not clearly stated in the main text, these values were obtained via tables, additional files, or by contacting the respective authors, if needed. All the extracted data were organized into master tables to facilitate cross-study comparability and narrative synthesis.

Table 2 summarizes the methodological and clinical variabilities of the 21 research papers included in this systematic review. Most studies used an observational methodology, primarily case control or cross-sectional, and focused on PCOS-specific populations or unselected IVF cohorts. FF was the most widely studied biological matrix, although numerous research also included miRNA profiling in GCs or EVs isolated from FF, which provided mechanistic insight into intrafollicular communication. The bulk of the research used RT-qPCR or TaqMan-based assays to quantify miRNAs, with a lower percentage using global profiling approaches, such as microarrays or NGS. Variability was seen in both the ovarian stimulation regimens utilized (e.g., GnRH agonist or antagonist COS) and the outcome measures studied. These spanned from molecular goals (such as miRNA–target pathway relationships) to clinical outcomes, like embryonic quality, oocyte maturity, and pregnancy rates.

### 2.6. Risk of Bias and Quality Assessment

To verify this systematic review’s credibility and methodological rigor, all the included observational studies were evaluated for risk of bias and research quality using the Newcastle–Ottawa scale (NOS). The NOS is a widely used and validated tool for assessing non-randomized studies, notably cohort and case-control designs, based on three core domains: research group selection, group comparability, and exposure or outcome assessment. The highest possible score is nine points, with higher scores signifying better methodological quality.

Each study was analyzed separately by two reviewers who used a predesigned score form based on NOS recommendations. In the domain of the selection, each study was evaluated for the representativeness of the exposed cohort (e.g., women with PCOS), the adequacy of the selection of the control group (e.g., normo-ovulatory IVF patients), the clarity and objectivity of the exposure definition (such as the source and handling of the FF, EVs, or GCs), and whether the outcome (e.g., IVF success or oocyte quality) was clearly absent at the study’s initiation. The comparability domain assessed how well studies accounted for potential confounders, such as demographic- (e.g., age and BMI), endocrine- (e.g., AMH and FSH), and stimulation-related variables. This control could be implemented using matching, stratification, or multivariate analysis. Finally, studies in the domain of outcome/exposure assessment were evaluated based on the objectivity and reliability of miRNA detection methods (e.g., RT-qPCR and NGS), the clarity of the outcome definitions (e.g., clinical pregnancy), and the completeness of reporting on follow-up or missing data.

Each study was given a numerical score for each of the three dimensions, and the cumulative score defined the study’s overall risk-of-bias status. Studies receiving from seven to nine points were regarded as having a low risk of bias, indicating that they satisfied high-level criteria for methodological integrity. Studies with four to six points were deemed to have a moderate risk of bias, typically because of insufficient control for confounding variables or an ambiguous exposure assessment. Studies with a score of less than four were classified as having a significant risk of bias, typically because of serious methodological problems, such as poor sample description, inappropriate outcome definitions, or a lack of reproducibility.

Discrepancies in scores between the two reviewers were documented and thoroughly discussed until agreement was reached. When no agreement could be achieved, a third reviewer independently reviewed the work and issued the final verdict. Throughout the procedure, all the choices were documented to guarantee transparency and reproducibility in accordance with PRISMA principles. In addition to the NOS-based assessment, research using in vitro or ex vivo models that did not work with the NOS tool were evaluated narratively. These research studies were evaluated based on experimental design criteria, such as replication, sample origin, clarity of miRNA extraction techniques, and the robustness of the downstream analysis. Although these studies could not be rated numerically, they were included in the synthesis because of their mechanistic importance and contribution to the molecular understanding of miRNA–pathway interactions in the follicular context.

Table 3 provides a thorough summary of the risk-of-bias evaluation, including the individual domain scores, overall NOS score, and final categorization for each included study. This organized method of quality assessment promotes openness and allows the reader to judge the strength and reliability of the information presented in connection with IVF outcomes and PCOS pathogenesis.

Table 3 contains the full breakdown of the quality assessments of the included research. The majority of the observational and case-control studies had scores from six to eight points, indicating a low-to-moderate risk of bias. Studies with less than four points were classified as high risk because of substantial methodological flaws, such as unclear selection criteria or insufficient control for confounding variables. In vitro experimental investigations were eliminated from formal NOS scoring but included in the synthesis for their mechanistic contributions. This assessment gives a critical context for assessing the dependability and strength of evidence from various study designs.

### 2.7. Data Synthesis and Analysis

Given the variability of the study designs, sample types, patient demographics, miRNA detection systems, and reported outcomes, a formal meta-analysis was deemed as inappropriate. As a result, the findings were organized and interpreted methodically using a narrative synthesis methodology. The synthesis sought to detect patterns of miRNA dysregulation in FF, GCs, or EVs and link them to IVF results and PCOS-specific characteristics. All the extracted data were initially classified based on the biological sample type (FF, GCs, or EVs) to highlight compartment-specific expression profiles. Within each sample group, studies were further classified according to demographic characteristics (PCOS vs. normo-ovulatory controls) and study objectives (clinical outcomes vs. mechanistic/pathway focus). This thematic grouping enabled this review to address both the descriptive and mechanistic aspects of miRNA function in the follicular milieu.

Each study recorded differentially expressed miRNAs, as well as their directionality (up- or downregulation), reported statistical significance (e.g., *p*-value, FDR, or log2FC), and reported any effect sizes (e.g., AUC). When available, relationships between miRNA levels and clinical indicators were investigated, including oocyte maturity, embryonic grading, blastocyst development, implantation, and pregnancy. Studies that reported validated or anticipated miRNA gene targets were thoroughly assessed, particularly when combined with pathway analysis (e.g., using KEGG, GO, or miRWalk).

The consistency of the miRNA findings across the research was evaluated qualitatively. MiRNAs that were differentially expressed in at least two independent investigations were labeled as “recurrently identified” and deemed as higher-priority candidates for further research. Discrepancies between the findings of the research were investigated in terms of sample discrepancies, stimulation protocol variations, and technical heterogeneity in miRNA detection methodologies.

To help with the synthesis, summary tables were constructed to consolidate the major findings from several investigations. Table 4 shows the consistently reported miRNAs, the direction of the dysregulation in PCOS vs. control groups, their known or projected targets, and the linked metabolic pathways. Where relevant, the potential clinical importance is explored, particularly for miRNAs associated with IVF results. Although quantitative synthesis was not attainable, frequency counts and the trend identification enabled evidence clustering. This approach preserved the dataset’s biological diversity while allowing for the organized analysis of very varied evidence.

Table 4 contains a thorough, integrated review of the most consistently reported miRNAs from the 21 research papers included, providing an in-depth understanding of their regulatory activities in the ovarian follicular environment. Each miRNA is identified by its dysregulation direction (upregulated or downregulated), the clinical or experimental context in which it was discovered (e.g., PCOS vs. control and IVF success or failure), validated or predicted molecular targets, and the biological signaling pathways in which it is involved. The table also includes clinical correlations that link miRNA expression patterns to important reproductive outcomes, such oocyte quality, embryonic development, implantation, pregnancy rates, and particular PCOS symptoms (e.g., hyperandrogenism, anovulation, and metabolic dysfunction).

This thorough table demonstrates the extensive network of post-transcriptional regulation mediated by miRNAs in granulosa cells, follicular fluid, and EVs, emphasizing their ability to fine-tune cellular activities essential for reproductive success. For example, miRNAs, like miR-132, miR-320, and miR-224, have been demonstrated to influence genes involved in estradiol synthesis and the FSH response, such as IGF1R and CYP19A1, hence altering oocyte maturation and follicular viability. MiR-146a and miR-155 regulate inflammatory pathways, including NF-κB, and have been linked to poor embryonic quality and implantation, particularly in PCOS.

By including signaling cascades, such as PI3K/AKT, TGF-β/SMAD, WNT/β-catenin, MAPK/ERK, and NF-κB, the table not only catalogs molecular targets but also stresses larger pathway abnormalities that contribute to the pathophysiology of infertility. These interactions demonstrate how miRNAs control various stages of follicular development, including hormonal responsiveness, angiogenesis, cell cycle regulation, and apoptosis. This table provides a conceptual framework for understanding how miRNAs behave as master regulators of ovarian function and reproductive potential.

Importantly, several of the miRNAs shown in the table have been found in multiple independent investigations, indicating their repeatability and biomarker potential. Their connections with quantifiable clinical outcomes, including the fertilization rate, blastocyst formation, and biochemical or clinical pregnancy, point to potential diagnostic or therapeutic applications in ART settings.

Table 4 summarizes the translational importance of miRNA studies in human reproduction. It connects molecular data to clinical findings and recommends high-priority candidates for further validation in large-scale cohorts. These miRNAs could be a new generation of indicators that improve IVF customization, aid in oocyte and embryonic selection, and deepen our understanding of PCOS-related infertility at the molecular level.

## 3. Results

The initial database search yielded 24,439 records, including 193 from PubMed, 1946 from Scopus, and 22,300 from WoS. Following automated and manual duplication removal (n = 190) and the first rejection of irrelevant publication genres, such as reviews, conference abstracts, case reports, and animal-only studies (n = 24,126), a total of 123 records were left for title and abstract screenings. Following screening, 88 articles were removed because they did not match the inclusion criteria or did not answer the review question. The entire texts of the remaining 35 records were obtained for eligibility determination. Four full-text papers were selected because they were not available in English, and another ten were excluded owing to insufficient methodological information, a lack of relevant outcome data, or the use of non-follicular samples. Finally, 21 papers passed all the eligibility requirements and were included in the final qualitative synthesis. Figure 1 visually summarizes the detailed selection procedure in accordance with the standards for PRISMA 2020 flow diagrams.

Figure 1 depicts the whole flow of the study selection process, guaranteeing transparency and reproducibility in compliance with the PRISMA 2020 principles. It emphasizes the broad reach of the initial search, the rigorous multi-stage screening procedure, and the reasons for article inclusion and deletion. The inclusion of 21 original papers indicates a carefully curated database that satisfied all the predefined criteria for population size, sample type, miRNA methodology, and outcome relevance to PCOS and IVF.

### 3.1. Differential MiRNA Expression in Women with vs. Without PCOS

Among the 21 studies considered, 15 focused on miRNA expression in women with polycystic ovarian syndrome (PCOS), either in contrast to normo-ovulatory controls or in IVF populations stratified by endocrine or metabolic characteristics. Several miRNAs were consistently dysregulated in follicular compartments, including follicular fluid (FF), granulosa cells (GCs), and extracellular vesicles (EVs) produced from FF, indicating a repeatable molecular hallmark of PCOS-related follicular dysfunction.

The downregulation of miR-132, miR-320, and miR-19b was shown to be frequent in PCOS patients’ FF and GCs. These miRNAs are known to influence genes implicated in the PI3K/AKT and IGF1R pathways, which are critical for follicular development, estradiol production, and oocyte maturation. MiR-320, in particular, adversely controls IGF1R, and inhibiting it may result in a poor FSH response and lower E2 production. This is consistent with the prevalent PCOS phenotypes of low intrafollicular E2 and anovulation. Similarly, miR-132 targets transcription factors involved in the LH response and steroidogenesis; its continuous downregulation in PCOS might indicate impaired luteinization and granulosa cell function.

In contrast, many studies found considerable elevation of proinflammatory and stress-related miRNAs, such as miR-146a, miR-155, and miR-21. These miRNAs affect the NF-κB- and TLR-signaling pathways, contributing to persistent low-grade inflammation, a common feature of PCOS. Elevated expression of miR-146a, in particular, has been associated with TRAF6/IRAK1 suppression, resulting in chronic inflammatory reactions inside the follicle, which may impair oocyte competence.

MiR-222 and miR-93 were also discovered to be elevated in PCOS populations. MiR-222 targets the estrogen receptor gene ESR1, and overexpression may lead to estrogen resistance in GCs, which is a crucial determinant in follicular arrest. MiR-93, on the other hand, regulates insulin signaling and has been associated with hyperinsulinemia and abnormal glucose metabolism in PCOS patients. Its increased expression lends credence to the biological link between metabolic imbalance and reproductive failure.

Importantly, numerous investigations found that miRNA dysregulation in PCOS was phenotype specific. For example, hyperandrogenic PCOS phenotypes (HA-PCOSs) have different miRNA profiles compared to normoandrogenic or lean PCOS subtypes. In these situations, miR-21, miR-27a, and miR-103 were more significantly dysregulated, indicating a link between androgen excess, oxidative stress, and follicular stoppage. This variability suggests that miRNAs not only reflect the existence of PCOS but also may distinguish across subtypes, possibly acting as tools for precision diagnosis and therapy stratification. Furthermore, certain miRNAs displayed different patterns according to the sample type or stage of follicular development. For example, miR-155 was observed to be enhanced in FF-derived EVs but not in GCs, indicating compartment-specific regulation or packing of miRNAs in the ovarian environment.

Table 5 highlights the core collection of miRNAs that have been consistently identified as dysregulated in PCOS compared to normo-ovulatory controls. These microRNAs have critical roles in granulosa cell biology, steroid hormone production, cell cycle regulation, and inflammatory signaling. Their altered expression patterns correspond to key pathophysiological aspects of PCOS, such as hyperandrogenism, insulin resistance, follicular arrest, and poor oocyte maturity.

Downregulated miRNAs, such as miR-132 and miR-320, have been associated with lower FSH sensitivity and estradiol production via the IGF1R- and CREB-signaling pathways, resulting in a poor follicular response. In contrast, increased miRNAs, such as miR-146a, miR-155, miR-222, and miR-93, are linked to inflammation, estrogen receptor repression, and insulin-signaling disruption. These miRNAs are hypothesized to contribute to the chronic inflammatory and endocrine–metabolic imbalances associated with PCOS, impacting both the follicular microenvironment and the clinical presentation of infertility.

### 3.2. MiRNAs Associated with Oocyte and Embryonic Qualities

Several research papers included in this review investigated the function of miRNAs in oocyte competence and embryo developmental potential during IVF. The qualities of the oocyte and subsequent embryo are critical predictors of ART success, driven by the dynamic interaction of internal molecular signals and external variables generated from the follicular milieu. MiRNAs, as key post-transcriptional regulators, have emerged as crucial components in this regulatory network.

One of the most regularly reported miRNAs linked with good outcomes is miR-202-5p, which is detected at high levels in FF and FF-derived EVs from women who produce mature oocytes (MII stage) and top-grade embryos. MiR-202-5p controls LHCGR, a receptor that is essential for ovulation induction and cumulus growth. It also affects BMP15 and GDF9, two important oocyte-secreted factors that regulate oocyte–cumulus cell interaction. Upregulation of miR-202-5p appears to promote synchronized cytoplasmic and nuclear maturation, possibly via increased luteinization and mitochondrial activity inside the oocyte.

In contrast, high levels of miR-27a, miR-21, and miR-23a have been linked to low oocyte quality and impaired embryonic development. These microRNAs play roles in cell survival, apoptotic signaling, and oxidative stress responses. For example, miR-27a inhibits SOD2 and BCL2, creating a pro-apoptotic follicular milieu. Its overexpression has been associated with early developmental arrest and decreased blastulation. Similarly, miR-21, although well-known as a survival factor in many tissues, appears to have context-dependent effects on the ovary. In excess, it may impede GC proliferation and alter normal follicle–oocyte signaling, reducing cytoplasmic maturation and embryonic viability.

Several studies found substantial relationships between miR-146a and embryonic quality. MiR-146a inhibits TRAF6 and IRAK1, altering TLR/NF-κB signaling and perhaps impairing GC function. It was first identified in the setting of inflammation. High levels of miR-146a in FF or EVs have been linked to fragmented embryos, low cleavage rates, and abnormal blastocyst shape, especially in PCOS and hyperinflammatory follicular settings.

Notably, miR-320a, miR-193b, and let-7 family members (especially let-7a and let-7f) have been associated with improved reproductive results. These miRNAs help follicular coordination by modulating genes, including IGF1R, MAPK1, and HMGA2, which promote granulosa cell proliferation, hormone synthesis, and cytoskeletal dynamics. Multiple studies found that greater levels of miR-320a were related with improved embryonic shape and implantation potential, indicating that it might be used as a noninvasive biomarker of oocyte quality. Some microRNAs have stage-specific interactions. For example, miR-92a and miR-103a have been linked to better cleavage-stage embryonic development but not blastocyst formation, whereas miR-24 and miR-30a appear to influence later stages of embryogenesis, possibly by regulating mitochondrial biogenesis and epigenetic remodeling during compaction.

Table 6 rovides a study-specific summary of the miRNAs most commonly related with oocyte and embryonic qualities. The table connects each miRNA to its proven or projected targets, altered biological pathways, and particular developmental consequences, bridging the gap between molecular profiling and clinical embryology. These findings support miRNAs’ use as indicators for determining embryonic potential and guiding oocyte selection in IVF.

### 3.3. MiRNAs Predictive of IVF Outcomes

Several studies in this review revealed distinct miRNAs, in FF, EVs, and GCs, which were strongly linked with critical IVF outcomes, such as the fertilization rate, blastocyst enlargement, implantation success, and verified clinical pregnancy. These miRNAs regulate a variety of biological pathways inside the follicular niche, and their extracellular release into FF or EVs makes them promising candidates for noninvasive biomarkers.

Zhang et al., 2017 found that miR-320a was highly elevated in FF samples from pregnant women [6]. This miRNA binds IGF1R and enhances FSH-induced steroidogenic activity in GCs via the PI3K/AKT pathway. Its enhanced expression was associated with improved oocyte maturity, higher-quality embryos, and greater implantation potential, indicating positive involvements in follicular competence and endometrial crosstalk. Santonocito et al., 2014 and Caponnetto et al., 2021 found that miR-202-5p was a good predictor of clinical pregnancy when raised in EVs. This miRNA regulates LHCGR and BMP15, facilitating cumulus–oocyte contact and maturation through TGF-β signaling. Its elevation in EV cargo in successful IVF cycles emphasizes its role in intercellular communication throughout the peri-ovulatory period [29,40].

Zhao et al., 2021 and Battaglia et al., 2020 found that miR-146a and miR-155 levels were considerably higher in the FF of patients with negative β-hCG findings [43,44]. MiRNAs have a role in inflammatory signaling via TRAF6/IRAK1 and the NF-κB axis, which can harm follicular integrity and embryo–endometrium communication. Elevated miR-146a levels in non-implanting individuals were related with altered cytokine gradients, indicating a microinflammatory state that is incompatible with a receptive endometrium.

Martinez et al., 2018 observed that miR-29a levels were consistently raised in non-pregnant women after embryonic transfer. As a regulator of extracellular matrix remodeling and endometrial adhesion molecules, its increased expression may signal endometrial resistance to embryonic invasion [46]. Similarly, Moreno et al., 2015 found that miR-200b downregulation was associated with ZEB1-mediated EMT dysregulation, leading to reduced GC-to-oocyte communication and a lower PR. Some research used ROC analysis to examine miRNAs as independent predictors [31]. Sørensen et al., 2016 found miR-223 to have an AUC of 0.81 for PR prediction [48], while Cui et al., 2021 found that low miR-125b in FF was related to significantly increased CPR (*p* = 0.04). These miRNAs were also supported by multivariate models that included E2, P, and AMH levels [41].

Table 7 summarizes the miRNAs found, in the 21 included studies, to be associated with IVF results. The table includes the direction of the expression, reported research, verified or projected molecular targets, and clinical interpretations. Notably, miR-320a, miR-202-5p, and miR-125b have been linked to improved fertilization success, embryonic quality, and clinical pregnancy, most likely through the regulation of the IGF1R-, LHCGR-, and c-MYB-signaling pathways. MiRNAs such as miR-146a, miR-155, and miR-29a, on the other hand, have regularly been linked to implantation failure or inferior embryonic development, most likely because of their participation in inflammatory or anti-adhesion pathways. A handful of miRNAs, including miR-21, miR-27a, and miR-133b, were found to be higher in poor-quality follicles or in PCOS-related subfertility, which might indicate larger disturbances in GC proliferation, oxidative stress, and hormonal feedback loops. Several of these miRNAs (e.g., miR-223 and miR-125b) were confirmed using ROC or statistical analysis, demonstrating their prognostic utility in clinical IVF situations.

### 3.4. Recurrent MiRNAs and Pathway Integration

A comparative synthesis of the 21 research papers identified a subset of miRNAs reported in several independent datasets. These recurrently dysregulated miRNAs show comparable expression patterns in both IVF patients with and without PCOS, and they appear to converge mechanistically on critical biological pathways involved in folliculogenesis, oocyte maturation, steroidogenesis, inflammation, and embryonic survival.

The most often mentioned and functionally important miRNAs were miR-202-5p, miR-320a, miR-21, miR-146a, miR-155, and let-7a. Their concentration in core intracellular signaling cascades underlines their potential as post-transcriptional reproductive process orchestrators. For example, miR-320a binds IGF1R and modifies the PI3K/AKT-signaling pathway in granulosa cells, increasing FSH sensitivity and promoting estradiol production. The upregulation of miR-320a has been linked to higher fertility rates and embryonic quality in IVF patients. MiR-202-5p, discovered as abundant in follicular extracellular vesicles from individuals who achieved pregnancy, controls LHCGR and BMP15, facilitating cumulus–oocyte communication via TGF-β/SMAD-mediated pathways and increasing nuclear oocyte maturation.

In contrast, miR-146a and miR-155 have been associated with poor IVF results and implantation failure, especially in PCOS and inflammatory phenotypes. These miRNAs reduce TRAF6, IRAK1, and SOCS1, activating the TLR/NF-κB axis and promoting inflammation in the follicular milieu. Their increase is associated with worse embryonic quality, poorer endometrial receptivity, and an overall unfriendly environment for implantation.

MiR-21 appears to perform a dual role. Although typically regarded as cytoprotective, high levels in the follicle have been associated with disturbed cumulus expansion and aberrant follicular development. MiR-21 affects cell survival, differentiation, and death in granulosa cells by modulating PI3K/AKT and MAPK signaling via PTEN and BCL2. Let-7a, another regularly reported miRNA, targets HMGA2 and has been linked to high-grade blastocyst development. It is thought to have roles in chromatin remodeling and cell cycle control, regulating embryonic development both before and after conception. Additional miRNAs, like miR-29a and miR-200b, although not the most common overall, provide insight into the possible interaction between follicular miRNA expression and uterine receptivity. These miRNAs influence extracellular matrix remodeling and epithelial-to-mesenchymal transition via modulating genes, including ZEB1, implying that they may facilitate embryo–endometrium interaction beyond the oocyte.

These miRNAs converge on signaling hubs, such PI3K/AKT, TGF-β/SMAD, NF-κB, MAPK/ERK, and WNT/β-catenin, highlighting their crucial function in regulating reproductive competence. Rather than operating independently, these miRNAs appear to create a regulatory network that controls granulosa cell activity, oocyte quality, hormonal response, and implantation potential. Their persistent presence in follicular fluid and extracellular vesicles bolsters their potential as noninvasive biomarkers and therapeutic targets in assisted reproduction.

Table 8 shows how recurrent miRNAs function as important molecular hubs in and across major signaling pathways. Their combined regulatory effects indicate that targeting a small subset of these miRNAs may be sufficient to impact a wide variety of reproductive activities. This functional convergence not only confirms their clinical importance but also provides a molecular basis for the development of miRNA-based diagnostics or therapeutics in ART.

## 4. Discussion

This SR emphasizes the critical significance of miRNAs as post-transcriptional regulators in the ovarian microenvironment, as well as their prognostic relationship with ART results. Consistent changes in miRNA expression were found in the FF, GCs, and EVs of IVF patients, including those with PCOS and normo-ovulatory profiles, according to the synthesis of the 21 research papers.

The results indicate that miRNAs function as active molecular mediators rather than passive FF components. Many target critical signaling hubs, such as PI3K/AKT, TGF-β/SMAD, NF-κB, MAPK/ERK, and WNT/β-catenin, which govern GC viability, oocyte maturation, hormonal sensitivity, and cytokine modulation. Dysregulation of these cascades, caused by abnormal miRNA profiles, may disturb the synchronization necessary for competent oocyte development, embryonic growth, and successful implantation.

The most reproducible miRNAs across the datasets were miR-320a, miR-202-5p, miR-21, miR-146a, and miR-155. MiR-320a promotes FSH-mediated steroidogenesis in GCs through IGF1R and PI3K signaling, whereas miR-202-5p regulates LHCGR and BMP15 via the TGF-β axis to influence oocyte–cumulus interactions. MiR-146a and miR-155 suppress TRAF6 and IRAK1 and, so, activate NF-κB, leading to microinflammation and poor follicular or endometrial health. MiR-21, although traditionally cytoprotective, may hinder cumulus growth when overexpressed via PI3K/BCL2 regulation.

The epigenetic–post-transcriptional link identified by Bhingardeve et al., 2025 provides significant mechanistic similarities to miRNA-related dysregulation observed in the papers included in this SR. Their findings on PCOS GCs demonstrated that promoter DNAme affects miRNA loci’s activities, with the hyper-DNAmes of miR-10b-5p, miR-127-3p, miR-23a-3p, and others suppressing miRNA output and derepressing apoptosis and stress-related targets, such as PTEN, MMP13, APAF1, and TET3 [11]. This regulatory logic is consistent with our discoveries in works such as those by Moreno et al., 2015 and Machtinger et al., 2017, in which miR-21 and miR-200b were connected to the regulation of the PI3K and EMT pathways, respectively, both downstream of PTEN and ZEB1 [31,34].

Bhingardeve et al. 2025, also discovered a hypo-DNAme for miR-140-5p and miR-200b-5p, which resulted in the overexpression and functional repression of WNT targets (FZD6 and LRP6) [11]. This molecular cascade is consistent with Moreno et al., 2015’s findings, which showed that miR-200b downregulation in FF was linked with lower blastocyst quality, implying that methylation-regulated miRNA fluctuation contributes directly to embryonic competence [31]. Similarly, miR-182-3p, raised in the Bhingardeve et al., 2025 cohort via hypo-DNAme [11], shares signaling overlap with both miR-155 (Zhao et al., 2021) and miR-146a (Battaglia et al., 2020), which were elevated in non-implanting patients in our SR. All of these converge on inflammation-prone NF-κB and oxidative pathways [43,44].

The epigenetic oversight of miRNAs targeting PTEN and ZEB1, presented in the Bhingardeve et al., 2025 dataset [11], supports our selection of miR-320a (Zhang et al., 2017) and miR-200b (Moreno et al., 2015) as pro-fertility regulators. In all circumstances, aberrant miRNA expression affects steroidogenic and transcriptional pathways that are necessary for GC health, oocyte maturation, and the E2 response [6,31]. Although our SR revealed miRNA dysregulation at the expression level, Bhingardeve et al., 2025 found that upstream methylomic reprogramming may be the primary driver, implying that the miRNA profiles observed in FF, GCs, and EVs across our studies may reflect deeper chromatin-level changes rather than isolated transcriptional variability [49].

Furthermore, the finding of the miRNA-DNAme crosstalk among PCOS symptoms provides an integrated paradigm in which both miRNA repression (e.g., miR-10b-5p → PTEN up) and activation (e.g., miR-140-5p → FZD6 down) affect folliculogenesis outcomes. This epigenetic axis is most likely regulated by the metabolic state, as evidenced by our included research (e.g., Caponnetto et al., 2021), in which EV-miRNA profiles were linked to CPR and embryonic quality in normo-ovulatory IVF [40]. The additional methylation component lends weight to the concept that miRNAs not only are effectors but also reflect upstream programming impacted by the PCOS ovary’s endocrine–epigenetic environment [50].

Cui et al., 2024 describe molecular data that corroborate the metabolic sensitivity of GCs in PCOS, which is mediated by EV-derived miRNA activity. Their work found higher levels of miR-34a-5p in FF-EVs from PCOS patients and established its capacity to target LDHA, an important enzyme in aerobic glycolysis. The miR-34a-5p suppression of LDHA impairs pyruvate–lactate conversion, depletes ATP, and causes death in KGN cells [51]. This mechanism backs up and expands on the metabolic disruption models proposed by our included studies, particularly Caponnetto et al., 2021, Zhao et al., 2021 and Battaglia et al., 2020, which found dysregulated miRNAs in FF-EVs associated with implantation failure, oxidative imbalance, and low-quality embryos [40,43,44].

Zhao et al., 2021 found that miR-155 and miR-146a were associated with NF-κB activation and enhanced GC apoptosis [43]. Cui et al., 2024 expand this paradigm by introducing a metabolic miRNA, miR-34a-5p, which converges on energy metabolism and ROS buildup via LDHA suppression [51]. Although Battaglia et al., 2020 focused on immunity-related miRNAs, both datasets show mechanistic convergence on GC death, indicating that several stressors—immune, oxidative, and metabolic—converge via miRNA-mediated pathways [44]. Furthermore, the involvement of LDHA, targeted by miR-34a-5p, provides a metabolic context for the findings of Moreno et al., 2015 and Cui et al., 2021, which linked glycolytic gene expression and oocyte maturation quality to changed FF miRNA levels [31,41].

Notably, none of the 21 papers in our SR specifically addressed LDHA targeting, although several—including those by Machtinger et al., 2017 and Naji et al., 2018—found lower ATP generation, GC proliferation problems, and altered apoptotic markers associated with miR-21 and miR-133b. This shows that miR-34a-5p acts as a parallel, but hitherto unknown, glycolytic gatekeeper, linking cellular metabolism and follicular competence [34,36]. Interestingly, the apoptotic phenotype seen with miR-34a-5p is similar to those of miR-27a (Xue et al., 2018) and miR-125b (Cui et al., 2021), all of which are included in our SR and have been connected to mitochondrial malfunction and BCL2-signaling suppression. Cui et al. 2021’s new findings do not contradict, but rather complement, the previously documented miRNA–apoptosis axis, emphasizing the multifaceted susceptibility of PCOS follicles [37,41].

Furthermore, the incorporation of the miR-34a-5p into FF-EVs supports the hypothesis—supported by Santonocito et al., 2014 and Scalici et al., 2016—that miRNA-containing EVs operate as mediators of intercellular stress signaling in the follicular niche. It is possible that miR-34a-5p, produced by stressed GCs, inhibits glycolysis in adjacent cells, perpetuating follicular atresia in a self-reinforcing cycle [29,32].

The ferroptotic axis reported by Lv et al., 2025 provides a unique and very significant mechanistic contribution to miRNA-mediated GC dysfunction in PCOS. Their in vivo and in vitro models revealed that serum EXOs from PCOS mice lacked miR-128-3p, resulting in increased expression of its direct target, CSF1, and the subsequent activation of p38/JNK/NRF2 signaling. This resulted in the suppression of SLC7A11, a glutamate–cystine antiporter and an essential ferroptosis checkpoint, causing lipid ROS buildup and iron overload in GCs. The subsequent loss of both mitochondrial integrity and cell viability connected this regulatory loop to decreased follicular survival and ovulatory failure [52].

Although miR-128-3p was not specifically studied in the 21 papers included in our SR, significant parallels can be established between miRNAs with similar effects on redox balance and GC apoptosis. In studies by Zhao et al., 2021 and Battaglia et al., 2020, high levels of miR-155 and miR-146a were linked to enhanced GC apoptosis through NF-κB and oxidative signaling [43,44]. Lv et al., 2025 have now extended this paradigm by demonstrating that the lack of a protective miRNA—miR-128-3p—can similarly affect GC integrity via a unique, but related, process involving ferroptosis rather than apoptosis [52]. The functional inhibition of SLC7A11 seen in the Lv et al., 2025 model [52] is similar to the BCL2/BAX dysregulation reported by Machtinger et al., 2017, where miR-21 levels were associated with altered survival signaling in immature follicles. In both cases, miRNA imbalance changed the intracellular milieu toward irreversible cell death—whether by traditional apoptosis or iron-dependent necrosis—highlighting GCs’ sensitivity to post-transcriptional stress [34].

Additionally, Lv et al., 2025 demonstrated that restoring miR-128-3p by agomiR injection directly improved the ovarian shape, E2 levels, and ovulatory phenotype in letrozole-induced PCOS mice [52]. This in vivo rescue is consistent with data obtained by Zhang et al., 2017 and Caponnetto et al., 2021, who found that favorable FF miRNA profiles (e.g., miR-320a and miR-202-5p) predicted oocyte competence and CPR [6,40]. Therefore, miR-128-3p appears as a missing regulatory element in the PCOS miRNA landscape—potentially a part of the miRNA’s “protective core” that promotes GC homeostasis, comparable to the involvements of miR-125b and miR-200b in FF-EVs [31,46].

The targeted pathway—p38/JNK/NRF2/SLC7A11—is noticeably absent from most clinical IVF-focused miRNA studies, despite intersecting with oxidative pathways reported by Scalici et al., 2016 and Cui et al., 2021. In both cases, mitochondrial depolarization and higher ROS levels were detected in low-quality follicles, but upstream regulators were not identified [32,41]. Lv et al., 2025’s latest findings fill this gap by discovering an miRNA-regulated ferroptotic switch inherent in GC physiology. From a translational standpoint, the EV-mediated deficit of miR-128-3p suggests that circulating or FF-EV miRNA screening might detect early molecular signs of follicular oxidative collapse, long before histological atresia or oocyte aneuploidy become apparent [52]. In this context, the miR-128-3p axis complements the miR-27a-3p/miR-15a-5p-GC dysfunction connections observed by Voros et al., 2025, establishing a panel of miRNAs associated with redox and metabolic stability [53].

Wyse et al., 2023’s findings shed light on how obesity affects the EV-miRNA cargo in FF, affecting the antral follicle’s signaling environment. These scientists discovered that obese and lean PCOS patients have varied levels of miRNA enrichment in FF-EVs targeting important pathways, including p53, FOXO, Hippo, MAPK, JAK/STAT, and TNF signaling. These mechanisms are known to regulate GC apoptosis, immune cell recruitment, and stress responses inside the ovarian niche. Several of these miRNAs were predominantly packed into FF-EVs in obese PCOS patients, whereas long non-coding RNAs were predominant in GCs. This demonstrates a compartment-specific transcriptomic response to metabolic stress. Importantly, the selective abundance of miRNAs that regulate apoptosis and proliferation in FF-EVs suggests that the follicle employs EV production as a compensation strategy to counteract intrafollicular oxidative and metabolic imbalances [54].

This inference substantially supports our SR findings, notably, those of Santonocito et al., 2014 and Caponnetto et al., 2021, who found that miR-202-5p, miR-320a, and other FF-EV miRNAs were linked with oocyte competence and CPR [29,40]. However, Wyse et al., 2023 introduced the feature of BMI stratification, emphasizing that obese PCOS patients have a fundamentally different EV-miRNA cargo than lean counterparts, which was not previously explored in depth in our SR [54]. The Wyse et al., 2023 study’s enriched miRNAs also overlap with known pro-apoptotic regulators [54], including miR-155 and miR-146a (Zhao et al., 2021 and Battaglia et al. 2020), indicating that obesity may aggravate follicular inflammation by increasing the packing of these molecules into FF-EVs. Their presence in EVs may serve two functions: indicating damage and boosting survival in nearby follicles by altering p53-dependent checkpoints [43,44].

Furthermore, the selective release of EVs harboring miRNAs targeting the p53 and FOXO pathways is associated with mitochondrial dysfunction and reduced oxidative stress buffering, as demonstrated by Scalici et al., 2016, Cui et al., 2021, and Martinez et al., 2018 [46]. In previous experiments, abnormal mitochondrial gene expression was associated with poor blastocyst development; Wyse et al., 2023 now present a mechanistic vector—EVs—through which such dysfunction may be spread across cells [32,41,46,54].

The FF-EV landscape reported by Wyse et al., 2023 is also consistent with the compensatory miRNA signals [54] found by Habibi et al., 2022, who interpreted the overexpression of certain miRNAs as a follicular attempt to regulate meiotic development under PCOS [39]. Similarly, the expression of proinflammatory and apoptotic EV-miRNAs in obese people (Wyse et al., 2023) may indicate an effort by the follicle to externalize detrimental signals while preserving the core oocyte–cumulus complex. Wyse et al., 2023’s discovered pathways (JAK/STAT, TNF, and Hippo) were underrepresented in our SR’s core pathway map, which concentrated on PI3K/AKT, TGF-β/SMAD, NF-κB, and MAPK. The current findings imply that obese PCOS follicles may recruit alternate or secondary pathways via EV-miRNA regulation, explaining the varied IVF responses observed in metabolically different PCOS phenotypes [54].

Voros et al., 2025 provide a complete molecular viewpoint on how epigenetic changes, including DNAme and ncRNA expressions, influence ovarian function and ART results. Their synthesis revealed consistent links between GC-specific miRNA dysregulation and decreased OR, oocyte competence, and implantation potential. These scientists identified miR-27a-3p and miR-15a-5p as being associated with GC mitochondrial dysfunction, E2 dysregulation, and poor response to COS. These markers share molecular similarities with miRNAs, previously identified in our SR (e.g., miR-21, miR-125b, and miR-146a), which regulate PI3K, BCL2, and apoptotic signaling in FF and GCs [53].

Global hypo-DNAme in GCs from low-responder and PCOS patients supports findings by Scalici et al., 2016 and Sørensen et al., 2016, who found altered FF-miRNA signatures in patients with decreased E2 production and inadequate blastocyst yields [32,48]. Voros et al., 2025 confirmed that DNAme aberrations affect miRNA expression by silencing protective loci or activating proinflammatory ones [53], paralleling the miRNA–gene deregulation axis observed by Bhingardeve et al., 2025 and supporting our SR’s inclusion of apoptosis-regulating miRNAs, such as miR-21, miR-27a, and miR-155 [49]. Moreover, their inclusion of histone modifications as a co-factor in miRNA expression stability helps to explain the expression-level miRNA results in studies by Moreno et al., 2015 and Naji et al., 2017, where changed miRNA abundance was detected but no underlying chromatin-state data were available. Voros et al., 2025 address this conceptual gap by identifying epigenetic programming as an upstream regulator of transcriptional and post-transcriptional activities [31,35,53].

Song et al., 2019 give more mechanistic clarification on the post-transcriptional control of the GC destiny in PCOS, identifying miR-186 and miR-135a as direct ESR2 repressors with downstream effects on CDKN1A, cell cycle progression, and apoptosis. These scientists found that both miRNAs were overexpressed in PCOS patients’ GCs and had positive correlations with serum E2. Luciferase tests indicated that ESR2 is a common target, and downregulation resulted in decreased CDKN1A expression, GC hyperproliferation, and apoptosis [55]. This trend corresponds to miRNA profiles published in various research studies in our SR. Machtinger et al., 2017 found that miR-21 overexpression led to PTEN downregulation and decreased apoptosis via PI3K/AKT [34]. Similarly, the Song et al., 2019 study found that miR-186/miR-135a → ESR2/CDKN1A leads to excessive GC proliferation and the loss of the regulatory checkpoint control. This shows that both miRNA families influence survival via different, but functionally comparable, signaling nodes [55].

Furthermore, the link between E2 levels and miR-186/miR-135a expression is consistent with the findings of Zhang et al., 2017 and Cui et al., 2021, who found that E2-responsive miRNAs in FF and EVs predicted oocyte maturity and follicular quality [6,41,56]. In the Zhao et al., 2021 dataset, higher miR-155 and miR-146a levels were connected to lower implantation rates, perhaps because of hormonal misregulation and NF-κB activation. This suggests that the hormone–inflammation–miRNA triangle may explain separate, but overlapping, molecular phenotypes [43]. The reduction of CDKN1A, a cyclin-dependent kinase inhibitor, adds to previous observations, by Naji et al., 2017 and Caponnetto et al., 2021, of the miRNA-induced dysregulation of cell cycle regulatory proteins in GCs. These changes, whether induced by miR-21, miR-125b, or, now, miR-186/135a, lead to aberrant proliferation patterns, which may explain follicular arrest and asynchronous oocyte–GC maturation dynamics [35,40]. Interestingly, although Song et al., 2019 focused on primary GCs and KGN models [55], the downstream targets and proliferation outcomes overlapped significantly with findings by Moreno et al., 2015 and Habibi et al., 2022, who linked FF-miRNA expression to GC metabolism and the apoptotic threshold. Together, these results support the notion that the GC destiny is regulated by a balance of miRNAs that promote or hinder survival, differentiation, and endocrine signaling [31,39].

The review by Duval et al., 2024 offers additional support for the functional importance of FF-derived EVs in reproductive pathology, with a focus on the selective packing and intercellular signaling activities of EV-encapsulated miRNAs in PCOS. These authors emphasize that FFEVs serve as a dynamic communication platform inside the follicular niche, replete with bioactive molecules, such as miR-379 and miR-200, which have previously been linked to female reproductive control [57]. Our study (Moreno et al., 2015; Martinez et al., 2018) identified miRNA-200 as a key regulator of EMT and cumulus–oocyte coordination through ZEB1 and TGF-β regulation. Their conclusion that EVs operate not only as carriers but also as functional regulators of follicular growth is consistent with mechanistic results from a number of included investigations [31,46]. Santonocito et al., 2014 and Scalici et al., 2016 showed that the FF-EV miRNA cargo predicted fertilization outcomes and CPR, whereas Caponnetto et al., 2021 connected miRNA patterns in FF-EVs to blastocyst quality and E2 levels. Duval et al., 2024 support this by suggesting that EV-associated miRNAs, such as miR-200, might serve as biomarkers and therapeutic agents in ART, which is consistent with our SR’s finding of miR-200b, miR-202-5p, and miR-320a as predictors of IVF success [29,32,40,57].

Duval et al., 2024’s investigation of EV heterogeneity in PCOS vs. endometriosis [57] supports findings by Zhao et al., 2021 and Cui et al., 2024, who found that aberrant EV-miRNA expression was related with poor implantation and GC stress. Their findings indicate that variations in the EV biogenesis and cargo may reflect greater follicular instability, which, in PCOS, is frequently exacerbated by metabolic and endocrine abnormalities [43,51]. These processes were also inferred by Wyse et al., 2023, who found that the EV-miRNA composition changed considerably with BMI status, altering pathways such as p53, TNF, and MAPK [54].

Furthermore, Duval et al., 2024’s notion that the EV content reflects the microenvironmental status of the follicle is consistent with the mechanistic theories provided in our SR: MiRNA enrichment or depletion within EVs can serve as a real-time readout of follicular health or approaching atresia [57]. This is confirmed by Habibi et al., 2022, who found that elevated miRNAs in FF-EVs compensated for GC damage, and Cui et al., 2024, who discovered that miR-34a-5p in EVs mediated glycolytic repression and GC apoptosis. Their comparison to endometriosis, albeit outside the scope of our SR, highlights the disease-specific tailoring of the EV cargo. The absence of EV overlap between PCOS and endometriosis implies that miRNA packaging is extremely context dependent, driven by unique inflammatory, hormonal, and metabolic profiles—offering the possibility of disease-specific biomarker panels [39,51].

## 5. Clinical Implications and Translational Prospects

The convergence of the findings from the 21 studies included in this SR, which is supported by recent high-impact investigations (e.g., by Bhingardeve et al., 2025, Cui et al., 2021, Lv et al., 2025, Wyse et al., 2023 and Duval et al., 2024), highlights the critical roles of miRNAs not only as passive biomarkers but also as active effectors of reproductive function in both PCOS and IVF populations. These tiny non-coding regulators function in a molecular hierarchy that integrates endocrine, metabolic, epigenetic, and immunological signals in the ovarian follicular niche [11,41,52,54,57].

MiRNAs—particularly those encased in FF-EVs—show excellent molecular stability, resistance to RNase degradation, and accessibility in clinical samples. This makes them suitable noninvasive indicators for assessing intrafollicular dynamics prior to follicular aspiration. Studies by Santonocito et al., 2014, Caponnetto et al., 2021 and Zhao et al., 2021 have shown that particular miRNA signatures (e.g., miR-320a, miR-202-5p, miR-200b, and miR-21) correspond with major ART outcomes, such as OR, the blastocyst rate, CPR, and LBR. Furthermore, the discovery of EV-associated miRNAs suggests a possible path for liquid-biopsy-based fertility diagnostics, especially in cases of cumulative cyclical failure, unexplained infertility, or poor-responder status [29,40,43].

These compounds have an equally deep translational potential. Several studies (e.g., those by Moreno et al., 2015, Cui et al. 2021 and Martinez et al., 2018) and recent investigations (e.g., those by Bhingardeve et al., 2025 and Lv et al., 2025) highlight the pathophysiological relevance of miRNAs targeting mitochondrial regulators (BCL2 and SLC7A11), metabolic enzymes (LDHA), signaling receptors (FSHR and ESR2), and key transcription factors (ZEB1 and PTEN) [31,41,46,49,52]. These findings pave the way for miRNA-based therapy techniques that leverage agomiRs, antagomiRs, and EV-mediated delivery systems to specifically control aberrant GC signaling in PCOS, poor responders, and women with recurrent implantation failures.

Furthermore, the epigenetic regulation of miRNAs, as demonstrated by Bhingardeve et al., 2025 and Voros et al., 2025 suggests that miRNAs may serve as molecular readouts of epigenetic imprinting, chromatin remodeling, and transcriptional potential, in addition to reflecting the follicle’s current functional state [49,53]. This makes them ideal for including in prediction models of ART success, particularly when paired with known indicators, like AMH, AFC, and E2.

In therapeutic contexts, the use of miRNA panels might provide:

- Early-stage embryonic selection using follicular miRNA profiling;
- Personalized COS procedures adapted to miRNA-regulated circuits;
- Cyclic classification in PCOS patients using inflammatory, metabolic, or epigenetic miRNA indicators;
- Adjunct-monitoring technologies that are used to evaluate the molecular impacts of adjuvant medications (such as metformin, antioxidants, and inositol) or lifestyle changes on follicular health.

Preclinical models developed by Cui et al., 2021 (miR-34a-5p) and Lv et al., 2025 (miR-128-3p) have demonstrated that targeted miRNA alteration may repair GC metabolism and vitality, restore normal folliculogenesis, and increase reproductive output [41,52]. This introduces a new class of molecularly directed ovarian treatments that may work in conjunction with oocyte rejuvenation approaches, stem-cell therapies, or epigenetic reprogramming. Finally, because EVs can cross the blood–follicle barrier and potentially enter the systemic circulation, their miRNA cargo may serve as both surrogate markers for ovarian function and long-term indicators of reproductive aging or endocrine disruption, expanding their clinical utility beyond IVF to broader gynecologic and endocrinologic practice.

Despite the promising insights provided by FF-derived miRNA profiling, several practical and translational challenges remain. First, the lack of a standardized protocol for the collection, handling, and storage of follicular fluid in IVF facilities introduces pre-analytical variability and adversely affects reproducibility. Variations in centrifugation, temperature regulation, and processing duration can significantly impact the stability and integrity of miRNAs, particularly in extracellular vesicles. Second, methodological variability obstructs cross-study comparisons and complicates assay harmonization, encompassing variations in EV isolation techniques (ultracentrifugation, precipitation, and filtration) and miRNA detection methodologies (RT-qPCR, microarrays, and NGS).

The clinical cost-effectiveness of multi-miRNA panels remains a significant challenge. Current high-throughput approaches remain impractical for widespread application in IVF facilities, particularly in the absence of the prior validation of a core panel of clinically predictive miRNAs.

Functional investigations are urgently required to elucidate the causal roles of miRNAs in ovarian physiology. Despite the establishment of several links, mechanistic investigations utilizing in vivo systems, oocyte maturation models, and granulosa cell cultures are essential. These should aim to validate direct gene targets, assess the phenotypic consequences of miRNA overexpression or inhibition, and determine if miRNAs are either genuine effectors of follicular malfunction and IVF failure or merely biomarkers.

A notable limitation among the incorporated studies is the considerable methodological diversity. Diversities in patient demographics (e.g., age, PCOS phenotype, and BMI), stimulation protocols (agonist versus antagonist), sample origins (whole follicular fluid, granulosa cells, or extracellular vesicles), and miRNA detection techniques (RT-qPCR, array based, or NGS) introduce variability that complicates inter-study comparisons. This synthesis is further complicated by the lack of standard outcome measurements, with some studies reporting embryonic grades, while others focus on biochemical pregnancy or cleavage rates.

The majority of the research encompassed in this evaluation possessed rather small sample sizes, thereby diminishing statistical power and increasing the probability of type II errors. This is particularly relevant for miRNAs like miR-224, which has been associated with increased estrogen synthesis and improved embryonic quality via the control of SMAD4 and the aromatase pathway. Despite being perhaps the most clinically relevant goal in ART, no prospective studies have yet established a direct correlation between miR-224 expression and live birth outcomes. Future multicenter, longitudinal studies employing consistent methodologies and adequate power are necessary to validate miR-224 and associated miRNAs as predictive biomarkers for IVF success.

In conclusion, miRNAs play a unique role at the junction of molecular diagnosis, pathway-based stratification, and targeted ovarian treatment. Their incorporation into ART workflows has the potential to shift fertility treatment away from a hormone-based empirical paradigm and toward a mechanism-driven, personalized-medicine approach that is consistent with the aims of precision reproductive medicine.

## 6. Limitations and Future Directions

Despite the quality and consistency of the data reported in this SR, a number of methodological and biological constraints must be recognized. First, the included publications’ research designs, which range from observational cohorts to in vitro studies, are heterogeneous, resulting in diversity in sample selection, miRNA quantification methodologies (e.g., RT-qPCR, microarrays, and NGS), and normalization procedures. This variety, although reflecting real-world research contexts, hampers straightforward cross-study comparisons and meta-analytic synthesis. Second, sample sources differed significantly between the research papers. Some miRNAs were examined in isolated GCs, whereas others were examined in complete FF and EV fractions. Although each compartment provides distinct insights into follicular physiology, the absence of consistency in EV separation, GC purification, and FF-handling techniques may affect miRNA stability and expression levels. Furthermore, the compartmentalization of miRNAs between intracellular pools and EV cargo, although physiologically significant, is not systematically addressed, limiting our understanding of their origin and function. A third issue is the lack of functional validation in many investigations. Although multiple articles identified miRNA-mRNA interactions using in silico approaches (e.g., TargetScan and miRDB), only a portion of these interactions were verified using luciferase assays, western blotting, or rescue studies. As a result, the mechanistic results of many investigations are associative rather than causal.

From a clinical viewpoint, most research papers were exploratory, with no long-term follow-up on live-birth outcomes, pregnancy problems, or offspring health. Many people used CPR or embryonic morphology as surrogate measures for success, which, although significant, may not fully convey ART efficacy. Furthermore, few studies controlled for confounding factors, like BMI, insulin resistance, or androgen levels, especially in PCOS populations, which may have a significant impact on both miRNA expression and reproductive outcomes. Furthermore, most studies are geographically and ethnically homogeneous, limiting generalization. The bulk of the studies were conducted in East Asia or Southern Europe, and cross-population heterogeneity in miRNA expression—because of environmental, genetic, or nutritional factors—has received little attention. This is particularly important for incorporating miRNA biomarkers into global clinical practice.

Despite its shortcomings, this SR provides a framework for further research. Prospective, multicenter, well-controlled research employing standardized miRNA extraction and EV methods, along with deep-sequencing and single-cell techniques, will be required to map the real miRNA landscape across follicular compartments. Combining multiomics platforms (e.g., transcriptomics, methylomics, and proteomics) with miRNA profiling may provide integrated signals predictive of oocyte competence, endometrial receptivity, and embryonic survival. Functionally, a deeper examination of miRNA-mRNA–protein networks, employing GC-deletion-specific mice, organoids, or ex vivo follicular cultures, may reveal actionable targets for therapeutic interventions. Equally crucial is the creation of miRNA-based classifiers for predicting ART outcomes, which should be tested across many populations and IVF systems.

Finally, the translational potential of miRNA therapies is unexplored. Building on preclinical successes (e.g., miR-128-3p agomiRs and miR-34a-5p inhibitors), clinical trials evaluating the safety, specificity, and efficacy of EV-based miRNA delivery systems have the potential to unlock a new class of reproductive therapeutics aimed at restoring follicular health, improving OR, and enhancing embryonic implantation.

## 7. Conclusions

This systematic study examined the roles of miRNAs in the control of ovarian functions in both patients with and without PCOS and undergoing IVF, focusing on miRNAs’ functional significance as epigenetic and post-transcriptional regulators. A consistent theme emerged from the 21 included studies, which included observational cohorts, case-control analyses, and experimental designs, as well as newly published mechanistic investigations: MiRNAs act as molecular integrators, coordinating signaling pathways required for GC survival, oocyte maturation, folliculogenesis, and embryonic implantation.

MiRNAs, including miR-21, miR-320a, miR-200b, miR-125b, miR-155, and miR-146a, play critical roles in regulating PI3K/AKT, TGF-β/SMAD, WNT/β-catenin, NF-κB, and MAPK signaling, which affect follicular development and endometrial receptivity. Their dysregulation, whether caused by DNAme (as shown by Bhingardeve et al., 2025) or environmental and metabolic stresses (e.g., obesity and insulin resistance), leads to aberrant gene expression in GCs, altered oocyte–GC interactions, and disturbed hormonal responsiveness. These miRNA-mediated processes are intimately related to clinical characteristics, such as low OR, poor embryonic quality, inadequate CPR, and recurrent implantation failures [49].

The discovery that FF-EVs carry bioactive miRNAs, allowing intercellular communication inside the follicular niche, is particularly noteworthy. Studies by Santonocito et al., 2014, Caponnetto et al., 2021 and Wyse et al., 2023 show that the FF-EV cargo reflects metabolic and inflammatory conditions in PCOS and may predict IVF success [29,40,54]. These vesicles can serve as a platform for miRNA-based diagnostics, possibly providing a less intrusive way to evaluate follicular health and oocyte competency in real time. Recent mechanistic research (e.g., by Cui et al., 2021, and Lv et al., 2025) has expanded this understanding by tying certain miRNAs—such as miR-34a-5p, miR-128-3p, and miR-186—to metabolic dysfunction, ferroptosis, and hormonal resistance in GCs [41,52]. These investigations not only validate the findings of this SR but also identify miRNAs as potential therapeutic targets. The preclinical restoration of beneficial miRNAs via agomiRs, or transport via designed EVs, has shown promise in reversing GC harm, restoring mitochondrial and glycolytic functions and increasing the ovulatory capacity, thereby laying the groundwork for molecular fertility therapies.

Translationally, miRNA panels may soon be used in ART regimens alongside established markers, such as AMH and AFC. Such panels might help to stratify PCOS patients according to their inflammatory/metabolic condition, guide COS regimens, and predict embryonic viability more accurately than using morphological criteria alone. Furthermore, miRNA analysis may enable cyclical optimization in low-level responders or recurrent ART failure patients, developing a tailored, precision-based reproductive treatment approach.

To summarize, miRNAs are a diverse family of molecular regulators with far-reaching effects on human reproduction. Their combined position as biomarkers and functional modulators puts them at the forefront of diagnostic, prognostic, and therapeutic innovations in ART. As our understanding of the epigenetic and extracellular miRNA landscape grows, these small compounds are set to become critical tools in the transition from empirical to mechanism-guided reproductive therapy, thereby enhancing outcomes for patients with PCOS and beyond.

## Figures and Tables

**Table 1:** Inclusion and exclusion criteria for the study selection.

Category	Inclusion Criteria	Exclusion Criteria
Population	Women of reproductive age undergoing IVF treatment, including both PCOS and non-PCOS patients; studies with defined PCOS diagnosis based on Rotterdam or NIH criteria.	Non-human studies, male infertility, pediatric populations, menopausal women, and studies without clearly defined PCOS diagnostic criteria.
Sample Type	Follicular fluid (FF) from aspirated ovarian follicles during oocyte retrieval; granulosa cells obtained during IVF; exosomes isolated from FF.	Studies measuring miRNAs in serum, plasma, whole blood, cervical mucus, or tissues not derived from the follicular microenvironment.
Study Design	Original research articles: observational (cohort, case-control, cross-sectional), experimental (in vitro with GCs), including both clinical and laboratory-based studies.	Reviews, meta-analyses, editorials, letters, conference abstracts, case reports, or non-peer-reviewed publications.
Outcome Measures	Studies reporting associations between miRNAs and IVF outcomes (e.g., fertilization, embryo quality, pregnancy), oocyte quality, PCOS phenotype, hormonal levels, or follicular markers.	Studies that report miRNA expression without any link to reproductive, hormonal, or IVF-related outcomes.
Language and Accessibility	Studies published in English with full-text availability through PubMed, Scopus, or institutional access.	Non-English articles, or articles with inaccessible full text or insufficient data for extraction.
miRNA Detection Method	Use od validated platforms such as RT-qPCR, TaqMan arrays, microarrays, or high-throughput sequencing for detection and qualification of miRNAs.	Studies using purely bioinformatic predictions or without experimental validation of miRNA expression.

This table summarizes the specified eligibility criteria used to identify the studies included in this systematic review. The criteria were predetermined using the PECO framework and implemented consistently during the title/abstract and full-text screenings. Only the studies that met all the inclusion criteria and none of the exclusion criteria were included.

**Table 2:** Characteristics of the included studies evaluating miRNAs in follicular fluid.

Study	Country	Design	Sample Size	Population/Group(s)	Sample Type	Detection Method	IVF Protocol	Statistical Method	Outcome Focus
Sang et al., 2013 [28]	China	Observational + in vitro	48	24 PCOS/24 Controls	FF (vesicles + supernatant)	TaqMan qPCR + deep sequencing	rFSH	Student’s *t*-test	PCOS vs. Control; miRNA expression; In vitro GC response
Santonocito et al., 2014 [29]	Italy/Sweden	Observational	15	Healthy IVF patients	FF (exosomes)	TaqMan array	IVF	SAM analysis	IVF success prediction; FF EVs’ miRNA profiles
Roth et al., 2014 [30]	USA	Observational	—	IVF patients	FF	RT-qPCR	IVF	Fold change	miRNA expressions in FF and IVF patients
Moreno et al., 2015 [31]	Spain	Observational	30	IVF by age/oocyte quality	FF + granulosa cells	Microarray + RT-qPCR	IVF	ANOVA, *t*-test	IVF outcomes and GC function; hormonal associations
Scalici et al., 2016 [32]	France	Case control	121	91 NOR/30 PCOS	FF	RT-qPCR	ICSI	ROC, Mann–Whitney	PCOS vs. non-PCOS; GC/FF miRNA profiling
Sørensen et al., 2014 [33]	Denmark	Case control	70	49 PCOS/21 Controls	FF	qPCR array + RT-qPCR	IVF	ROC, Pearson correlation	PCOS vs. NOR; miRNA and oocyte qualities
Machtinger et al., 2017 [34]	Israel/USA	Observational	40	IVF patients	FF	TaqMan OpenArray (754 miRNAs)	IVF	Fold change, *p*-value	PCOS vs. Control; FF miRNA related to inflammation
Naji et al., 2017 [35]	Iran	Case control	—	PCOS/Controls	FF	RT-qPCR	IVF	*t*-test	Association of FF miRNA with embryonic quality
Naji et al., 2018 [36]	Iran	Case control	—	PCOS/Controls	FF	RT-qPCR	IVF	*t*-test	Differential FF miRNA in PCOS vs. Control
Xue et al., 2018 [37]	China	Observational	—	IVF patients	FF	RT-qPCR	IVF	Spearman correlation	Differential FF miRNA in PCOS vs. Control
Zhang et al., 2017 [6]	China	Case control	—	PCOS/Controls	FF	RT-qPCR	IVF	*t*-test	IVF outcome; FF miRNAs and oocyte quality
Zhang et al., 2021 [38]	China	Observational	—	PCOS/Controls	FF	RT-qPCR	IVF	*t*-test	PCOS metabolic profile; FF miRNA associations
Habibi et al., 2022 [39]	Iran	Case control	—	PCOS/Controls	FF	RT-qPCR	IVF	—	miRNA–mRNA correlations in FF; IVF outcomes
Caponnetto et al., 2021 [40]	Italy	Observational	—	IVF patients	FF (Exosomes)	RT-qPCR	IVF	—	FF exosome miRNAs in high- vs. low-quality oocytes
Cui et al., 2021 [41]	China	Observational	—	IVF (good vs. poor follicular quality)	FF	RT-qPCR	IVF	*t*-test	GC miRNAs and hormonal markers in IVF
Khan et al., 2021 [42]	Pakistan	Observational	—	IVF patients	FF	qPCR	IVF	—	FF miRNA signatures; embryonic and pregnancy outcomes
Zhao et al., 2021 [43]	China	Observational	—	IVF patients	FF	RT-qPCR	IVF	—	Oocyte maturity; FF miRNA signature
Battaglia et al., 2020 [44]	Italy	Observational	—	IVF patients	FF	RT-qPCR	IVF	*t*-test	PCOS vs. Control; miRNA and hormonal status
Qasemi et al., 2020 [45]	Iran	Experimental (in vitro)	—	PCOS model (granulosa cells)	GC	qPCR	—	—	Embryonic quality and IVF outcomes; FF miRNA
Martinez et al., 2018 [46]	Spain	Observational	—	IVF patients	FF	RT-qPCR	IVF	—	miRNA markers for IVF success
Liao et al., 2025 [47]	China	Observational	—	IVF patients	FF	RT-qPCR	IVF	—	miRNA profiles associated with PCOS and IVF

This table summarizes the main characteristics of the 21 original studies included in this systematic review. It includes information on the study design, population type, sample origin (FF, GCs, or EVs), miRNA detection platforms, IVF protocol specifics, and primary outcome emphasis. The research presented covers a wide range of analytical methods and sample types related to PCOS and ART outcomes.

**Table 3:** Quality assessment of the included studies using the Newcastle–Ottawa scale.

Study	Design	Selection (0–4)	Comparability (0–2)	Outcome/Exposure (0–3)	Total Score (0–9)	Risk of Bias
Sang et al., 2013 [28]	Observational + in vitro	3/4	1/2	2/3	7/9	Low
Santonocito et al., 2014 [29]	Observational	2/4	1/2	2/3	6/9	Low
Roth et al., (2014) [30]	Observational	2/4	1/2	1/3	5/9	Moderate
Moreno et al., 2015 [31]	Observational	3/4	1/2	2/3	7/9	Low
Scalici et al., 2016 [32]	Case control	4/4	2/2	2/3	8/9	Low
Sørensen et al., 2014 [33]	Case control	3/4	2/2	2/3	7/9	Low
Machtinger et al., 2017 [34]	Observational	2/4	1/2	2/3	6/9	Low
Naji et al., 2017 [35]	Case control	1/4	1/2	1/3	3/9	High
Naji et al., 2018 [36]	Case control	1/4	1/2	1/3	3/9	High
Xue et al., 2018 [37]	Observational	2/4	1/2	1/3	5/9	Moderate
Zhang et al., 2017 [6]	Case control	2/4	1/2	2/3	6/9	Low
Zhang et al., 2021 [38]	Observational	1/4	1/2	1/3	3/9	High
Habibi et al., 2022 [39]	Case control	2/4	1/2	2/3	6/9	Low
Caponnetto et al., 2021 [40]	Observational	2/4	1/2	1/3	5/9	Moderate
Cui et al., 2021 [41]	Observational	2/4	1/2	2/3	6/9	Low
Khan et al., 2021 [42]	Observational	1/4	-	1/3	2/9	High
Zhao et al., 2021 [43]	Observational	2/4	-	1/3	3/9	High
Battaglia et al., 2020 [44]	Observational	2/4	1/2	2/3	6/9	Low
Qasemi et al., 2020 [45]	Experimental (in vitro)	-	-	-	-	Not applicable
Martinez et al., 2018 [46]	Observational	2/4	1/2	2/3	6/9	Low
Liao et al., 2025 [47]	Observational	2/4	1/2	2/3	6/9	Low

This table presents the risk-of-bias evaluation for each included study using the NOS. Scores are provided for each category (selection, comparability, and outcome/exposure) and used to categorize the research as having a low, moderate, or high risk of bias. The NOS scores eliminated studies that used in vitro models and did not include human clinical data.

**Table 4:** Summary of differentially expressed miRNAs and their target genes, pathways, and clinical associations.

MiRNA	Validated Target Genes	Biological Function/Pathway	Clinical Relevance/Condition
miR-132	HMGA2, RAB5B	Regulates insulin sensitivity and follicular cell proliferation; enhances estradiol production via targeting of growth and trafficking pathways.	Polycystic ovary syndrome (PCOS); associated with poor follicular maturation when downregulated.
miR-320	IGF1R, TGFβ1	Involved in FSH-stimulated estradiol synthesis and oocyte development; modulates TGF-beta signaling affecting folliculogenesis.	Downregulated in PCOS; potential biomarker of oocyte competence in IVF.
miR-24	EGFR, CYP19A1	Suppresses estrogen synthesis by inhibiting aromatase (CYP19A1); may impair granulosa cell function and follicular development.	Reduced expression linked to low E2 levels in PCOS.
miR-222	ESR1, PTEN	Modulates estrogen receptor alpha (ERα) and insulin signaling via PTEN; may affect endometrial receptivity and GC proliferation.	Upregulated in PCOS; potential compensatory mechanism in insulin-resistant states.
miR-151-3p	BCL2L1	Targets anti-apoptotic BCL2L1 in granulosa cells; its downregulation promotes apoptosis, contributing to follicular atresia.	Downregulated in hyperandrogenic PCOS (HA-PCOS); AUC = 0.91 for phenotypic discrimination.
miR-29a	DNMT3A, COL4A1	Regulates extracellular matrix (ECM) and methylation patterns; important for the implantation window and embryo–endometrium interactions.	Downregulated in PCOS and linked to poor IVF outcomes.
miR-146a	IRAK1, TRAF6	Key regulator of inflammation via the NF-κB pathway; suppresses proinflammatory cytokines in the follicular environment.	Upregulated in PCOS and poor responders during IVF; linked to oxidative stress.
miR-202-5p	LHCGR	Promotes oocyte meiotic competence by modulating LH receptor signaling; enriched in high-quality oocytes.	Positive marker of fertilization and blastocyst development in IVF.
miR-766-3p	WNT5A	Involved in WNT-mediated embryonic patterning and trophoblast differentiation; influences the cleavage-stage embryonic morphology.	Upregulated in top-quality embryos in IVF cycles.
miR-224	CYP19A1	Enhances aromatase expression and estradiol biosynthesis; promotes granulosa cell proliferation and follicular growth.	Positively associated with successful IVF outcomes.
miR-27a	PPARγ, SREBP1	Alters lipid metabolism and steroid precursor synthesis; modulates follicular fluid composition and thecal cell function.	Overexpressed in PCOS; linked to follicular arrest and anovulation.
miR-34a	BCL2, SIRT1	Promotes apoptosis and reduces cell survival via mitochondrial pathways; is associated with oxidative imbalance.	Elevated in poor-quality FF in IVF cycles.
miR-93	CDKN1A, E2F1	Regulates cell cycle transition (G1/S); enhances follicular recruitment and proliferation of granulosa cells.	Differential expression in responders vs. non-responders in IVF.
miR-21	PDCD4, RECK	Prevents apoptosis and maintains GC viability; supports angiogenesis within follicles through matrix remodeling inhibition.	Associated with both PCOS inflammation and successful embryonic implantation.
miR-155	SOCS1	Controls immune cell activation and cytokine feedback loops; modulates the intrafollicular inflammatory milieu.	Impacts oocyte competence and IVF outcomes through immunomodulation.

This table summarizes the miRNAs discovered in the included studies, which have been shown to vary in expression in PCOS and IVF populations. It covers validated gene targets, linked biological pathways, and known or suspected clinical relevance. These miRNAs are important molecular regulators of the follicular environment, granulosa cell activity, and reproductive consequences.

**Table 5:** Differentially expressed miRNAs in PCOS vs. non-PCOS women.

MiRNA	Regulation in PCOS (↑/↓)	Validated or Predicted Targets	Associated Pathways	Molecular/Cellular Role(s)	Clinical Implications
miR-132	↓	IGF1R, CREB1	PI3K/AKT, Steroidogenesis, CREB signaling	Regulates GC differentiation, aromatase activity, and E2 production by modulating CREB-mediated transcription.	Low miR-132 may impair FSH-induced E2 synthesis, leading to anovulation and follicular arrest in women with PCOS.
miR-320	↓	IGF1R	FSH signaling, Folliculogenesis, MAPK	Enhances FSH sensitivity in GCs by regulating IGF1R expression and promotes oocyte competence.	Reduced miR-320 compromises follicular responsiveness to gonadotropins, contributing to poor IVF outcomes in PCOS.
miR-146a	↑	IRAK1, TRAF6	NF-κB, TLR signaling, Inflammatory responses	Acts as a negative feedback regulator in inflammatory signaling, modulating innate immunity pathways.	Elevated levels may reflect or exacerbate the chronic low-grade inflammation seen in PCOS follicles, impairing oocyte quality.
miR-155	↑	SHIP1, SOCS1	NF-κB, JAK/STAT	Controls immune cell activation, cytokine signaling, and GC survival under stress.	Associated with oxidative stress and immunity dysregulation in PCOS; may contribute to altered GC function and subfertility.
miR-222	↑	ESR1, CDKN1B	Estrogen signaling, Cell cycle	Suppresses ESR1, reduces estrogen responsiveness, and modulates cell proliferation in GCs.	Linked to estrogen resistance and abnormal follicular development common in hyperandrogenic PCOS phenotypes.
miR-93	↑	IRS1, CDKN1A	Insulin signaling, Cell cycle regulation	Modulates insulin receptor substrate and cell cycle inhibitors, integrating metabolic and reproductive signaling.	Upregulated in obese or insulin-resistant PCOS phenotypes; may impair GC proliferation and oocyte competence.

This table contains an enlarged synthesis of miRNAs that were shown to be differently expressed between women with and without PCOS in the included studies. It contains the direction of the expression (up/downregulation), verified or anticipated target genes, critical signaling pathways, cellular activities, and clinical implications for ovarian dysfunction, hormonal resistance, inflammation, and reproductive outcomes.

**Table 6:** MiRNAs linked to oocyte and embryonic qualities according to the included studies.

MiRNA	Regulation (↑/↓)	Reported in	Validated/Predicted Targets and Pathways	Developmental Stage Affected	Detailed Clinical Association
miR-202-5p	↑	Santonocito et al., 2014, Caponnetto et al., 2021 [29,40]	LHCGR, BMP15, TGF-β, and gonadotropin signaling	MII oocyte, blastocyst	↑ mature oocytes, improved blastocyst formation
miR-27a	↑	Xue et al., 2018, Khan et al., 2021, [37,42]	SOD2, BCL2, oxidative stress, and apoptosis	Cleavage stage	↑ fragmentation, ↓ cleavage rate
miR-21	↑	Machtinger et al., 2017 [34]	PTEN, BCL2, PI3K/AKT, and GC function	Oocyte maturation	↓ cumulus expansion, immature oocytes
miR-146a	↑	Zhao et al., 2021, Battaglia et al., 2020 [43,44]	TRAF6, IRAK1, and NF-κB	Embryonic morphology	↑ inflammatory signature, poor embryonic quality
miR-320a	↑	Zhang et al., 2017 [6]	IGF1R and FSH responsiveness	Fertilization, cleavage	↑ fertilization, better embryonic quality
let-7a	↑	Sang et al., 2013 [28]	HMGA2 and chromatin regulation	Blastocyst morphology	↑ advanced embryonic morphology
miR-133b	↑	Naji et al., 2017 [35]	–	–	associated with oocyte dysmaturity in PCOS
miR-200b	↓	Moreno et al., 2015 [31]	ZEB1 and EMT pathways	Oocyte–GC communication	↓ linked to suboptimal follicular function
miR-223	↑	Sørensen et al., 2016 [48]	–	–	Detected in the FF of poor-prognosis IVF patients
miR-29a	↑	Martinez et al., 2018 [46]	–	–	Upregulated in FF with low embryonic stem cell implantation rate
miR-125b	↓	Cui et al., 2021 [41]	–	–	Suggested as an indicator of oocyte competence
miR-150	↑	Roth et al., 2014 [30]	c-Myb and immunity and GC regulation	–	Positively associated with embryonic cleavage

This table highlights the major miRNAs found, in the included studies, to be connected with oocyte competence and embryonic development. It comprises the expression direction, literature references, molecular targets, involved pathways, altered embryonic development stage, and clinical significance.

**Table 7:** MiRNAs predictive of IVF outcomes according to the included studies.

miRNA	Regulation (↑/↓)	Reported in	Targets and Pathways	Associated IVF Outcome	Detailed Clinical Interpretation
miR-320a	↑	Zhang et al., 2017 [6]	IGF1R and PI3K/AKT	Implantation, CPR	↑ FF levels → ↑ fertilization, embryonic quality, and PR
miR-202-5p	↑	Santonocito et al., 2014, Caponnetto et al., 2021 [29,40]	LHCGR, BMP15, and TGF-β	Embryonic competence, PR	↑ in EVs of pregnant women; enhances maturation signaling
miR-146a	↑	Zhao et al., 2021, Battaglia et al., 2020 [43,44]	TRAF6, IRAK1, and NF-κB	Implantation failure	↑ FF expression → poor embryonic quality and local inflammation
miR-155	↑	Zhao et al., 2021 [43]	SOCS1 and immunity regulation	Implantation failure	↑ in non-pregnant patients, immunity activation and follicular stress
miR-29a	↑	Martinez et al. 2018 [46]	ECM remodeling	Negative implantation outcome	↑ FF expression associated with endometrial resistance
miR-200b	↓	Moreno et al., 2015 [31]	ZEB1 and EMT	Low PR	↓ levels impair GC–oocyte signaling and the follicular response
miR-223	↑	Sørensen et al., 2016, [48]	–	CPR (AUC = 0.81)	↑ FF expression with ROC-based prediction of PR
miR-125b	↓	Cui et al., 2021 [41]	c-MYB	CPR (*p* = 0.04)	↓ FF levels → ↑ PR, a potential marker of competence
miR-21	↑	Machtinger et al., 2017 [34]	PTEN and PI3K/AKT	Implantation potential	↑ in GCs with poor cumulus expansion and ↓ fertilization
miR-133b	↑	Naji et al., 2017 [35]	–	Oocyte maturation, PR	Linked to oocyte dysmaturity and IVF failure in PCOS
let-7a	↑	Sang et al., 2013 [28]	HMGA2	Embryonic morphology, PR	↑ associated with improved blastocyst development
miR-27a	↑	Xue et al., 2018 [37]	BCL2 and SOD2	Embryonic fragmentation	↑ associated with early developmental arrest

This table presents the miRNAs, reported in the included studies, to be associated with IVF outcomes, including implantation, fertilization, and pregnancy rates. It includes both directly and indirectly correlated miRNAs, with references to their regulation patterns, molecular targets, functional pathways, and detailed clinical interpretations. When full validation data were not available, interpretive insight was derived from experimental or FF/EV-associated expression profiles.

**Table 8:** Recurrent miRNAs, validated targets, molecular pathways, and functional implications.

MiRNA	Validated Target Genes	Biological Function/Pathway	Clinical Relevance/Condition
miR-132	HMGA2, RAB5B	Regulates insulin sensitivity and follicular cell proliferation; enhances estradiol production via the targeting of growth and trafficking pathways.	Polycystic ovary syndrome (PCOS); associated with poor follicular maturation when downregulated.
miR-320	IGF1R, TGFβ1	Involved in FSH-stimulated estradiol synthesis and oocyte development; modulates TGF-beta signaling affecting folliculogenesis.	Downregulated in PCOS; a potential biomarker of oocyte competence in IVF.
miR-24	EGFR, CYP19A1	Suppresses estrogen synthesis by inhibiting aromatase (CYP19A1); may impair granulosa cell function and follicular development.	Reduced expression linked to low E2 levels in PCOS.
miR-222	ESR1, PTEN	Modulates estrogen receptor alpha (ERα) and insulin signaling via PTEN; may affect endometrial receptivity and GC proliferation.	Upregulated in PCOS; potential compensatory mechanism in insulin-resistant states.
miR-151-3p	BCL2L1	Targets anti-apoptotic BCL2L1 in granulosa cells; its downregulation promotes apoptosis, contributing to follicular atresia.	Downregulated in hyperandrogenic PCOS (HA-PCOS); AUC = 0.91 for phenotypic discrimination.
miR-29a	DNMT3A, COL4A1	Regulates extracellular matrix (ECM) and methylation patterns; important for the implantation window and embryo–endometrium interaction.	Downregulated in PCOS and linked to poor IVF outcomes.
miR-146a	IRAK1, TRAF6	The key regulator of inflammation via the NF-κB pathway; suppresses proinflammatory cytokines in the follicular environment.	Upregulated in PCOS and poor responders during IVF; linked to oxidative stress.
miR-202-5p	LHCGR	Promotes oocyte meiotic competence by modulating LH receptor signaling; enriched in high-quality oocytes.	A positive marker of fertilization and blastocyst development in IVF.
miR-766-3p	WNT5A	Involved in WNT-mediated embryonic patterning and trophoblast differentiation; influences cleavage-stage embryonic morphology.	Upregulated in top-quality embryos in IVF cycles.
miR-224	CYP19A1	Enhances aromatase expression and estradiol biosynthesis; promotes granulosa cell proliferation and follicular growth.	Positively associated with successful IVF outcomes.
miR-27a	PPARγ, SREBP1	Alters lipid metabolism and steroid precursor synthesis; modulates follicular fluid composition and thecal cell function.	Overexpressed in PCOS; linked to follicular arrest and anovulation.
miR-34a	BCL2, SIRT1	Promotes apoptosis and reduces cell survival via mitochondrial pathways; associated with oxidative imbalance.	Elevated in poor-quality FF in IVF cycles.
miR-93	CDKN1A, E2F1	Regulates the cell cycle transition (G1/S); enhances follicular recruitment and the proliferation of granulosa cells.	Differential expression in responders vs. non-responders in IVF.
miR-21	PDCD4, RECK	Prevents apoptosis and maintains GC viability; supports angiogenesis within a follicle through matrix-remodeling inhibition.	Associated with both PCOS inflammation and successful embryonic implantation.
miR-155	SOCS1	Controls immune cell activation and cytokine feedback loops; modulates the intrafollicular inflammatory milieu.	Impacts oocyte competence and IVF outcomes through immunomodulation.

This table summarizes the most commonly reported miRNAs in the research, including their known or projected targets, participation in important signaling pathways (e.g., PI3K/AKT, NF-κB, and TGF-β), and functional roles in ovarian biology and reproductive outcomes.

## Data Availability

No new data were created or analyzed in this study.
